# Supplementary material for: A CSB-PAF1C axis restores processive transcription elongation after DNA damage repair
Source: Nat Commun. 2021 Feb 26;12:1342. doi: 10.1038/s41467-021-21520-w (PMC7910549; doi:10.1038/s41467-021-21520-w)
Supplement: Supplementary file 1 — Supplementary Information [file 41467_2021_21520_MOESM1_ESM.pdf]

Supplementary Information

A CSB-PAF1C axis restores processive transcription elongation after DNA damage repair

Diana van den Heuvel[1], Cornelia G. Spruijt[2,3†], Román González-Prieto[4†], Angela Kragten[1], Michelle T. Paulsen[5], Di Zhou[6], Haoyu Wu[1], Katja Apelt[1], Yana van der Weegen[1], Kevin Yang[5,7], Madelon Dijk[1], Lucia Daxinger[1], Jurgen A. Marteijn[6], Alfred C.O. Vertegaal[4], Mats Ljungman[5,8], Michiel Vermeulen[2], and Martijn S. Luijsterburg[1*]

Correspondence to: m.luijsterburg@lumc.nl

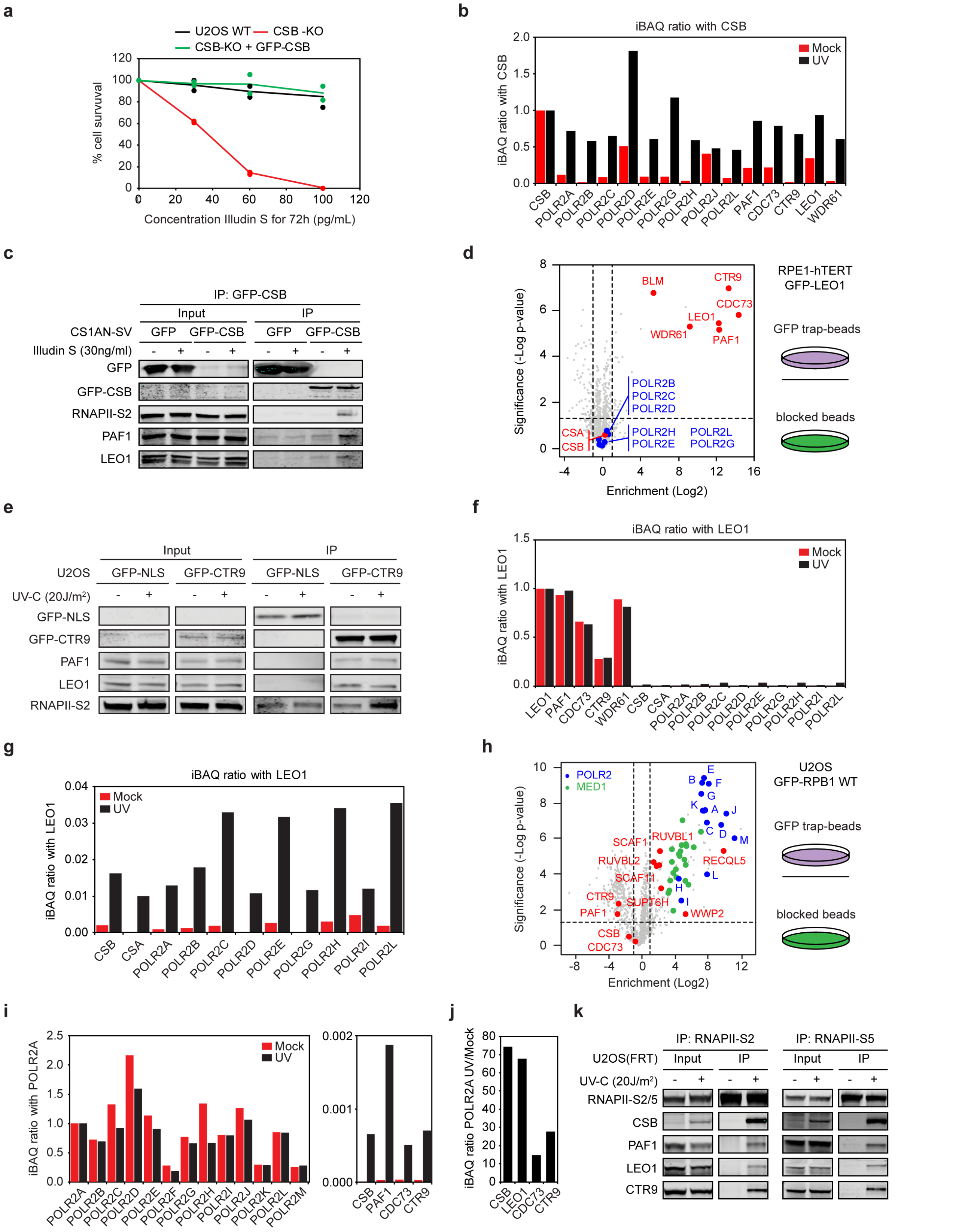
a
U2OS WT
CSB -KO
CSB-KO + GFP-CSB
% cell survuval
Concentration Illudin S for 72h (pg/mL)
b
iBAQ ratio with CSB
iBAQ ratio with CSB
Mock
UV
c
IP: GFP-CSB
Input
IP
CS1AN-SV
GFP
GFP-CSB
Illudin S (30ng/ml)
GFP
GFP-CSB
RNAPII-S2
PAF1
LEO1
d
Significance (-Log p-value)
Enrichment (Log2)
RPE1-hTERT GFP-LEO1
GFP trap-beads
blocked beads
e
Input
IP
U2OS
GFP-NLS
GFP-CTR9
UV-C (20J/m²)
RNAPII-S2
f
iBAQ ratio with LEO1
iBAQ ratio with LEO1
g
iBAQ ratio with LEO1
iBAQ ratio with LEO1
h
POLR2
MED1
Significance (-Log p-value)
Enrichment (Log2)
U2OS GFP-RPB1 WT
GFP trap-beads
blocked beads
i
iBAQ ratio with POLR2A
j
iBAQ ratio POLR2A UV/Mock
k
IP: RNAPII-S2
IP: RNAPII-S5
U2OS(FRT)
Input
IP
UV-C (20J/m²)
RNAPII-S2/5
CSB
PAF1
LEO1
CTR9

Supplementary Figure 1. Interactions between CSB – RNAPII – PAF1C

a Clonogenic Illudin S survival of WT, CSB-KO and reconstituted CSB-KO with GFP-CSB. Data represent mean and individual datapoints of two independent experiments. **b** Stoichiometry of the interactions of the indicated proteins with GFP-CSB in unirradiated (red) and UV-irradiated U2OS CSB-KO cells (black) based on the iBAQ values obtained from label-free MS. **c** Single co-immunoprecipitation of GFP or GFP-CSB from U2OS cells treated or untreated with 30 ng/ml Illudin S for 3 hours. **d** Volcano plot depicting the specific interactors of GFP-LEO1 after pulldown of GFP-LEO from RPE1-hTERT cells using GFP trap beads over blocked beads, analyzed by label-free MS. The enrichment (log^2) is plotted on the x-axis and the significance (2-sided t-test -log^{10} p-value) is plotted on the y-axis. Highlighted are important proteins with a known role in DNA repair including PAF1C, BLM, CSA and CSB (red) and RNAPII (blue). **e** Single co-immunoprecipitation of GFP-NLS or ectopically expressed GFP-CTR9 from U2OS cells with or without UV-induced DNA damage. **f** Stoichiometry of the interactions of the indicated proteins with GFP-LEO1 in unirradiated (red) and UV-irradiated RPE1-hTERT cells (black) based on the iBAQ values obtained from label-free MS. **g** Stoichiometry of the interactions of the indicated proteins with GFP-LEO1 in unirradiated (mock; red) and UV-irradiated U2OS cells (black) based on the iBAQ values obtained from label-free MS. **h** Volcano plot depicting the enrichment of proteins after pull-down of GFP-RPB1 from U2OS cells analyzed by label-free MS. The enrichment (log^2) is plotted on the x-axis and the significance (2-sided t-test -log^{10} p-value) is plotted on the y-axis. Highlighted are significantly enriched subunits of RNAPII (blue), mediator (green) and several known interactors of RNAPII (red). Note that CS proteins and PAF1C subunits are not detected in unirradiated cells under these conditions. **i** Stoichiometry of the interactions of the indicated proteins with POLR2A / RPB1 in unirradiated (red) and UV-irradiated cells (black) based on the iBAQ values obtained from label-free MS. **j** Fold enrichment in stoichiometry of the interactions of the indicated proteins with POLR2A / RPB1 after UV-irradiation over unirradiated condition, based on the iBAQ values obtained from label-free MS. **k** Co-immunoprecipitation of endogenous RNAPII-S2 (left; representative of at least n=3) or RNAPII-S5 (right; n=1) from U2OS cells in the absence or presence of UV-induced DNA damage.

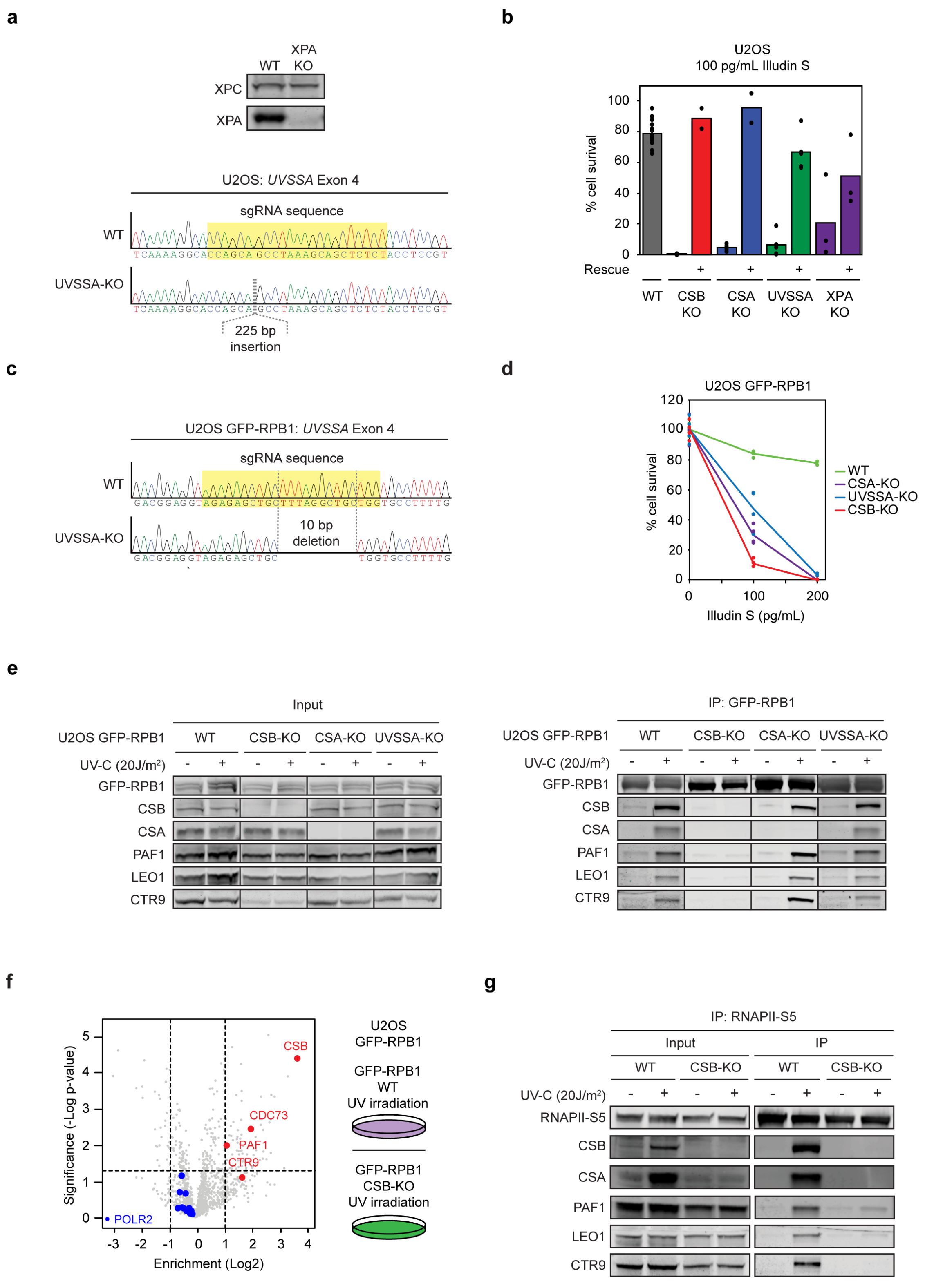
a
WT
XPA KO
XPC
XPA
U2OS: UVSSA Exon 4
sgRNA sequence
WT
UVSSA-KO
225 bp insertion
b
U2OS
100 pg/mL Illudin S
% cell surival
100
80
60
40
20
0
Rescue
+
WT
CSB KO
CSA KO
UVSSA KO
XPA KO
c
U2OS GFP-RPB1: UVSSA Exon 4
sgRNA sequence
WT
UVSSA-KO
10 bp deletion
d
U2OS GFP-RPB1
% cell surival
120
100
80
60
40
20
0
0
100
200
Illudin S (pg/mL)
WT
CSA-KO
UVSSA-KO
CSB-KO
e
Input
IP: GFP-RPB1
U2OS GFP-RPB1
WT
CSB-KO
CSA-KO
UVSSA-KO
UV-C (20J/m²)
- +
GFP-RPB1
CSB
CSA
PAF1
LEO1
CTR9
f
Significance (-Log p-value)
Enrichment (Log2)
CSB
CDC73
PAF1
CTR9
POLR2
U2OS GFP-RPB1
GFP-RPB1 WT UV irradiation
GFP-RPB1 CSB-KO UV irradiation
g
IP: RNAPII-S5
Input
IP
WT
CSB-KO
UV-C (20J/m²)
RNAPII-S5
CSB
CSA
PAF1
LEO1
CTR9

Supplementary Figure 2. CSB mediates the UV-induced RNAPII – PAF1C interaction

a Western blot to confirm the knockout of XPA (top; representative of n>3) and sequencing to confirm the knockout of *UVSSA* (bottom) in U2OS cells. **b** Clonogenic Illudin S survival of U2OS WT, CSA, CSB, UVSSA and XPA knockout cell lines and their rescue with corresponding GFP-tagged proteins at 100 pg/mL Illudin S. Data represent mean and individual data points of technical and/or biological replicate experiments. **c** Sequencing to confirm the knockout of *UVSSA* in U2OS GFP-RPB1 cells. **d** Clonogenic Illudin S survival of GFP-RPB1 WT, CSA, CSB, and UVSSA knockout cell lines at 100 or 200 pg/mL Illudin S. Data represent mean and individual data points of technical and/or biological replicate experiments. **e** Co-immunoprecipitation of GFP-RPB1 from U2OS WT, CSA, CSB, and UVSSA knockout cells in the absence or presence of UV-induced DNA damage. Representative figure of at least 2 experiments. **f** Volcano plot depicting the enrichment of proteins after pull-down of GFP-RPB1 from U2OS WT or CSB-KO cells after UV-induced DNA damage, analyzed by label-free MS. The enrichment (log_2) is plotted on the x-axis and the significance (2-sided t-test $-log_{10}$ p-value) is plotted on the y-axis. Highlighted are subunits of RNAPII (blue) and PAF1C and CSB (red). **g** Single co-immunoprecipitation of endogenous RNAPII using Ser5-specific antibodies from U2OS WT or CSB-KO cells in the absence or presence of UV-induced DNA damage.

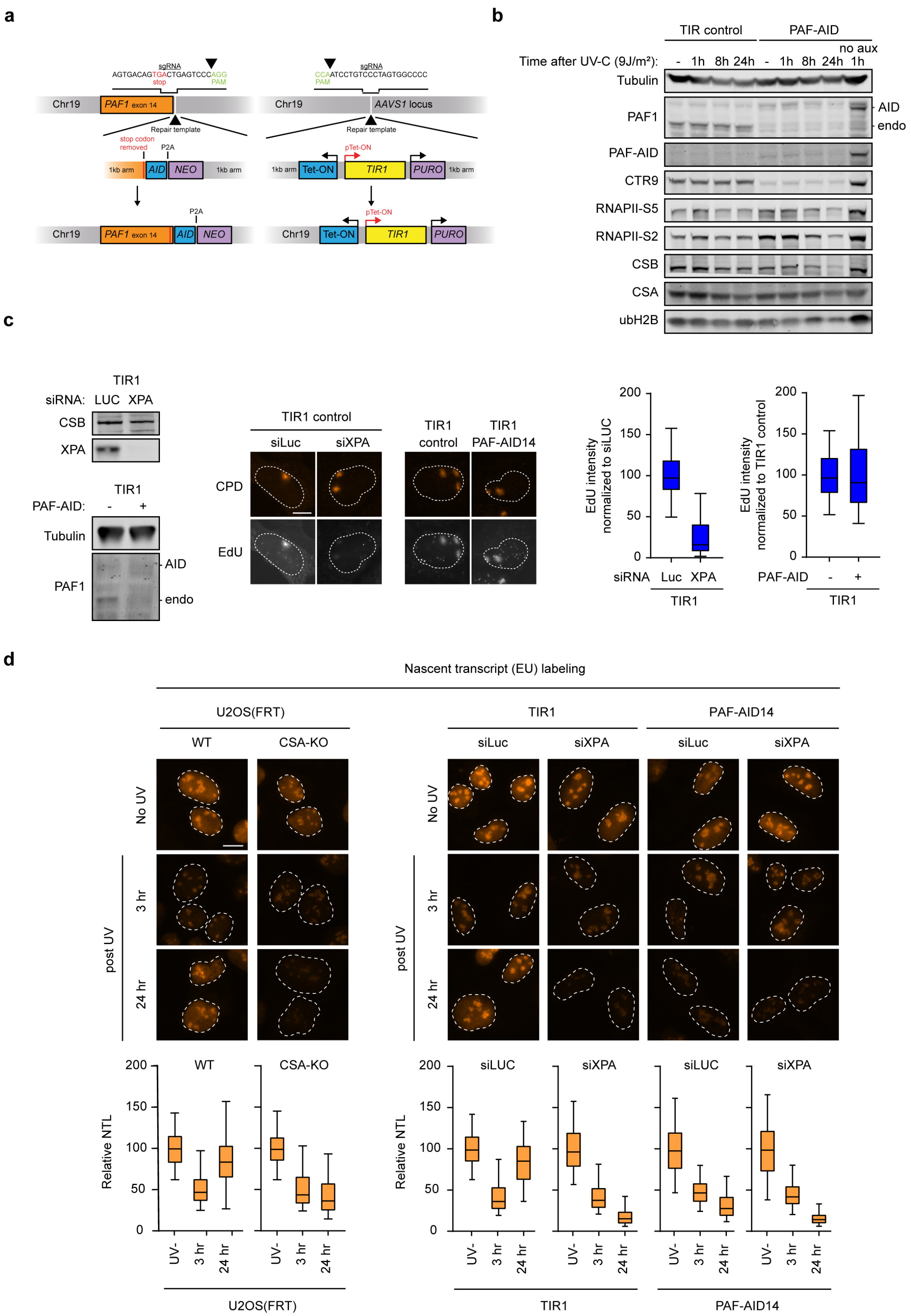
a
sgRNA
AGTGACAGTGACTGAGTCCCAGG
stop
PAM
Chr19
PAF1 exon 14
Repair template
stop codon removed
P2A
1kb arm
AID
NEO
1kb arm
CCAATCCTGTCCCTAGTGGCCCC
AAVS1 locus
pTet-ON
Tet-ON
TIR1
PURO
b
TIR control
PAF-AID
no aux
Time after UV-C (9J/m²): - 1h 8h 24h - 1h 8h 24h 1h
Tubulin
PAF1
AID
endo
PAF-AID
CTR9
RNAPII-S5
RNAPII-S2
CSB
CSA
ubH2B
c
TIR1
siRNA: LUC XPA
CSB
XPA
PAF-AID: - +
Tubulin
PAF1
TIR1 control
siLuc
siXPA
TIR1 control
PAF-AID14
CPD
EdU
EdU intensity normalized to siLUC
EdU intensity normalized to TIR1 control
siRNA Luc XPA
PAF-AID - +
d
Nascent transcript (EU) labeling
U2OS(FRT)
WT
CSA-KO
TIR1
PAF-AID14
siLuc
siXPA
No UV
post UV
3 hr
24 hr
Relative NTL
siLUC
UV-
U2OS(FRT)
TIR1
PAF-AID14

Supplementary Figure 3. PAF1-AID knockin cells are not involved in GGR, and controls for nascent transcription analyses

a Outline of the approach to generate PAF1-AID knockin cells. The DOX-inducible osTIR1 cassette was first targeted to the *AAVS1* locus. The AID-NEO cassette was subsequently targeted to the last exon of the endogenous *PAF1* locus whereby the stop codon was removed to generate a PAF1-AID in-frame fusion. **b** Western blot analyses of protein expression in total cell lysates of control U2OS TIR1 cells or PAF1-depleted U2OS PAF-AID cells at various timepoints after UV (9J/m^2). Representative figure of at least 2 experiments **c** Left; western blot to confirm XPA knockdown (top) and auxin-mediated depletion of PAF1 (bottom). Representative figures of 3 replicates. Middle; representative images of 5 ethynyl-deoxyuridine (5-EdU) labeling at local sites of DNA damage visualized by CPD staining in indicated condition. Scale bar indicates 10 µm. Right; quantification of the 5-EdU incorporation under the indicated conditions. Boxplots represent the median, 5th and 95th percentile of all cells of three independent experiments. **d** Representative images (top) and quantification (bottom) of 5-ethynyl-urdine (5-EU) incorporation in WT versus CSA-KO cells (left panels) or TIR1 versus PAF-AID1 cells treated with the indicated siRNAs and 5h auxin treatment before UV irradiation (right plots). Scale bar indicates 10 µm. Boxplots represent the median, 5th and 95th percentile of all cells of three independent experiments.

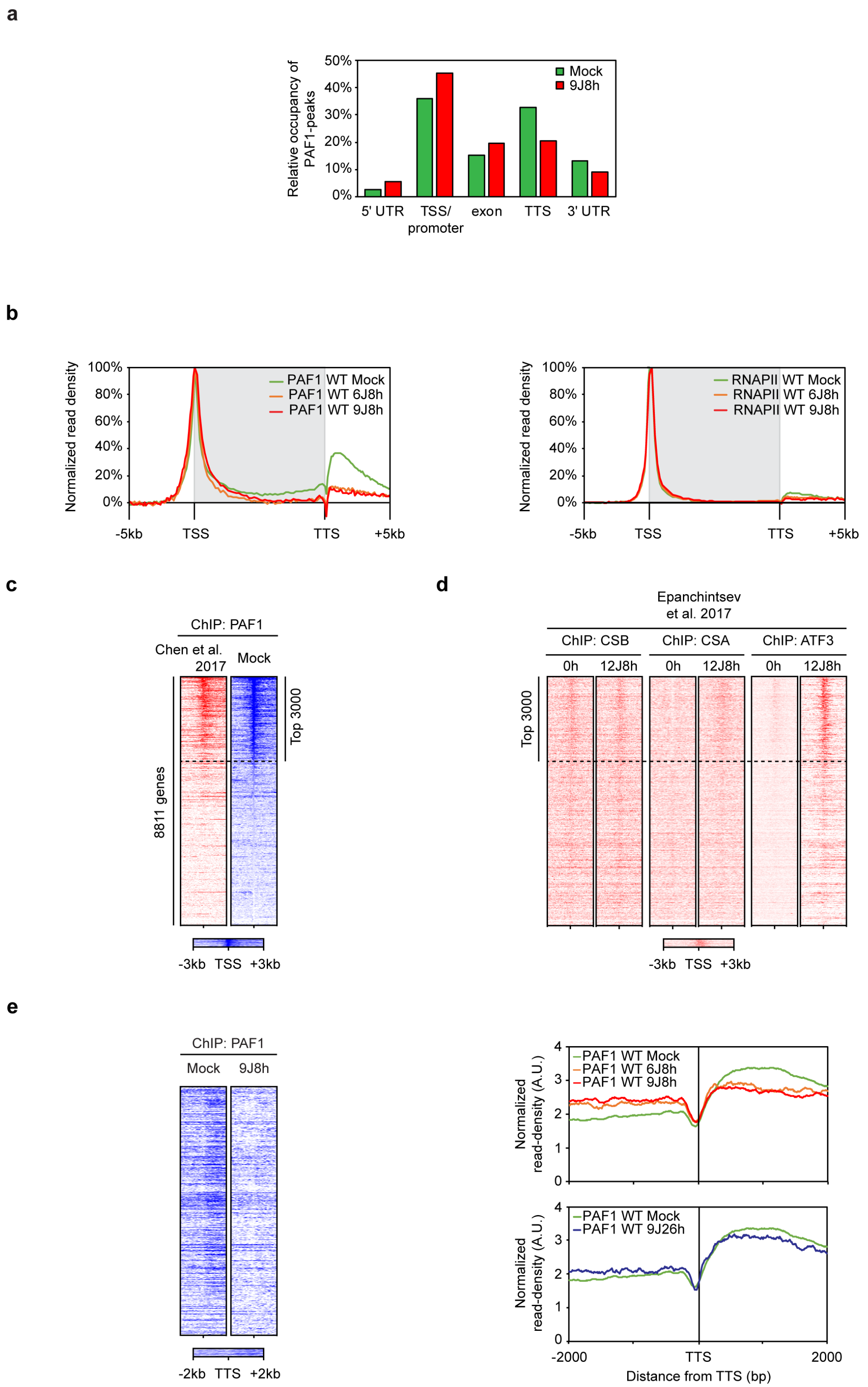
a
Relative occupancy of PAF1-peaks
50%
40%
30%
20%
10%
0%
Mock
9J8h
5' UTR
TSS/ promoter
exon
TTS
3' UTR
b
Normalized read density
100%
80%
60%
40%
20%
0%
PAF1 WT Mock
PAF1 WT 6J8h
PAF1 WT 9J8h
-5kb
TSS
TTS
+5kb
RNAPII WT Mock
RNAPII WT 6J8h
RNAPII WT 9J8h
c
ChIP: PAF1
Chen et al. 2017
Mock
8811 genes
Top 3000
-3kb TSS +3kb
d
Epanchintsev et al. 2017
ChIP: CSB
ChIP: CSA
ChIP: ATF3
0h
12J8h
Top 3000
-3kb TSS +3kb
e
ChIP: PAF1
Mock
9J8h
-2kb TTS +2kb
Normalized read-density (A.U.)
4
3
2
1
0
PAF1 WT Mock
PAF1 WT 6J8h
PAF1 WT 9J8h
PAF1 WT 9J26h
-2000
TTS
2000
Distance from TTS (bp)

Supplementary Figure 4. PAF1 occupancy throughout the genome and comparison of ChIP-seq data with published datasets

a Occupancy of PAF1 binding-peaks across the coding regions of the genome in unirradiated (mock) and UV-irradiated cells. Peaks were identified using MACS2 of sample over input after pooling reads of 3 mock and 3 UV-irradiated replicates. **b** Averaged metaprofile of PAF1 (left) or RNAPII (right) binding across the top 3,000 genes with PAF1 binding at the TSS as identified in c and Fig. 5d. Averaged data represent mock-treated cells (PAF1 n=3, RPB1 n=3), or cells 8h after UV-irradiation with 6 J/m^2 (PAF1 n=1, RPB1 n=3) or 9 J/m^2 (PAF1 n=3, RPB1 n=2). Profiles are normalized to 100% at TSS instead of area under the curve. **c** Heatmaps around the TSS from PAF1 ChIP-seq data of 8,811 genes ranked according to PAF1 signal at the TSS in unirradiated cells (mock; blue) of which the first 3,000 genes show strong PAF1 enrichment. Data is compared to publish data (in red). **d** Heatmaps of CSB, CSA and ATF3 ChIP-seq from the Egly lab around the TSS of 8,811 genes in unirradiated and UV-irradiated cells (8 hours after 12 J/m^2). Genes are ranked as in c and show the strongest binding of CSB, CSA and ATF3 in the top 3,000 genes identified in c. **e** Heatmaps and averaged metaplots from PAF1 ChIP-seq data around the TTS of the 3,000 genes with strong PAF1 binding (identified in c and Fig. 5d) in unirradiated cells (mock, n=3), in cells 8h after UV-irradiation with 6 J/m^2 (n=1) or 9 J/m^2 (n=3) and 26h after 9 J/m^2 (n=1).

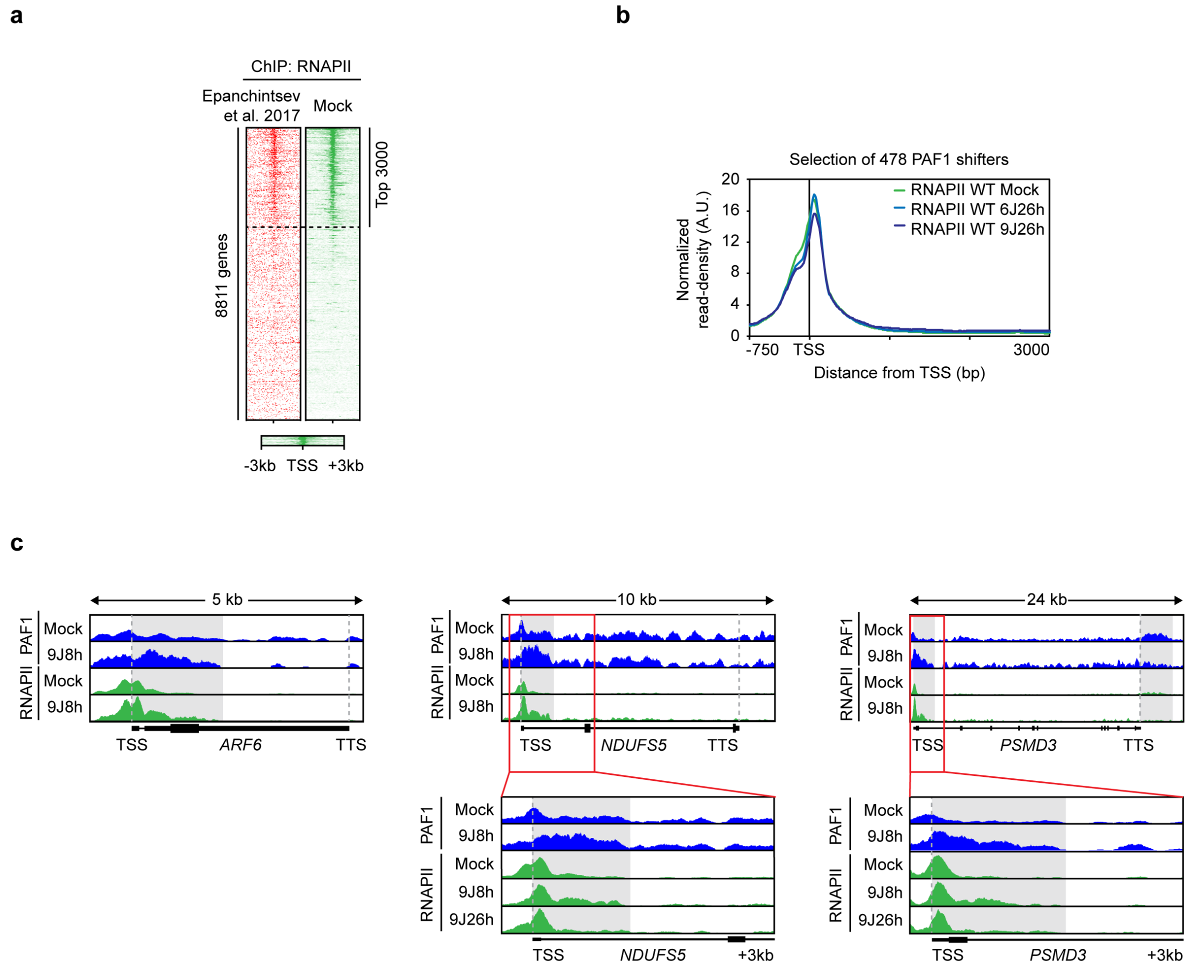
a
b
ChIP: RNAPII
Epanchintsev et al. 2017
Mock
Top 3000
8811 genes
-3kb TSS +3kb
Selection of 478 PAF1 shifters
RNAPII WT Mock
RNAPII WT 6J26h
RNAPII WT 9J26h
Normalized read-density (A.U.)
Distance from TSS (bp)
-750 TSS 3000
c
5 kb
10 kb
24 kb
PAF1
RNAPII
Mock
9J8h
9J26h
TSS
TTS
ARF6
NDUFS5
PSMD3
+3kb

Supplementary Figure 5. Comparison of ChIP-seq data with published datasets and distribution of PAF1 and RNAPII across several representative genes

a Heatmaps of genes as in Supplementary Figure 4c, but for RNAPII ChIP-seq data in unirradiated cells (mock; green), compared to publish data (in red). **b** Averaged metaplots of RNAPII ChIP-seq in unirradiated WT cells (mock, n=3) and UV-irradiated WT cells (26 hours after 6 J/m^2 (n=2) or 9J/m^2 (n=2)) for the 478 genes that show a consistent PAF1 right-shift after UV irradiation in all replicate ChIP-seq experiments (All Shift; Fig. 6a, c). **c** UCSC genome browser track showing the PAF1 and RNAPII read densities across the *ARF6* gene of ~5 kb (left), *NDUFS5* gene of ~10 kb (middle), and *PSMD3* gene of ~24 kb (right) in U2OS cells after pooling all replicate reads of the indicated conditions, as described in Table S6. Insets of the first 3 kb of each gene is shown at the bottom.

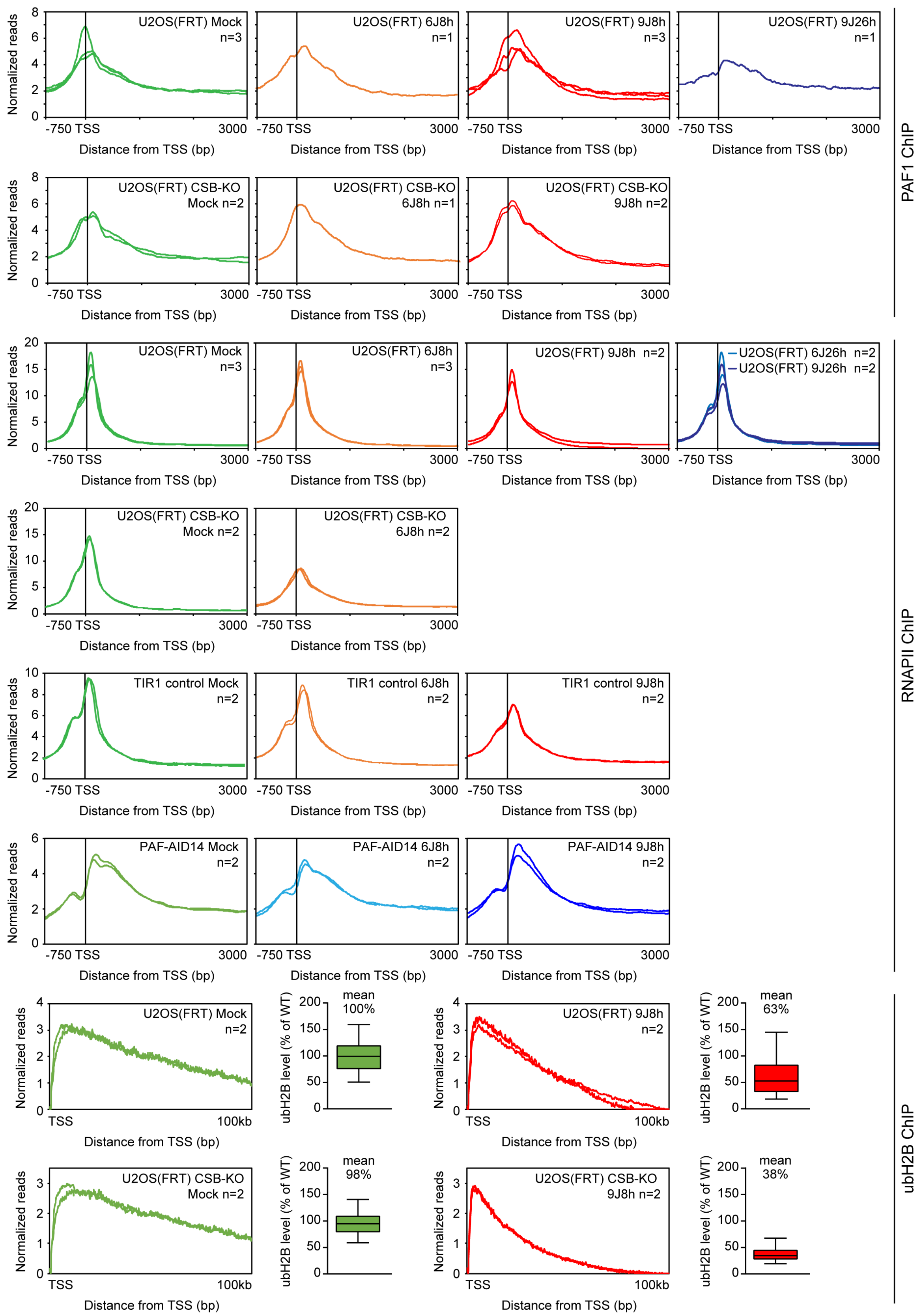
PAF1 ChIP
U2OS(FRT) Mock n=3
U2OS(FRT) 6J8h n=1
U2OS(FRT) 9J8h n=3
U2OS(FRT) 9J26h n=1
U2OS(FRT) CSB-KO Mock n=2
U2OS(FRT) CSB-KO 6J8h n=1
U2OS(FRT) CSB-KO 9J8h n=2
RNAPII ChIP
U2OS(FRT) Mock n=3
U2OS(FRT) 6J8h n=3
U2OS(FRT) 9J8h n=2
U2OS(FRT) 6J26h n=2
U2OS(FRT) 9J26h n=2
U2OS(FRT) CSB-KO Mock n=2
U2OS(FRT) CSB-KO 6J8h n=2
TIR1 control Mock n=2
TIR1 control 6J8h n=2
TIR1 control 9J8h n=2
PAF-AID14 Mock n=2
PAF-AID14 6J8h n=2
PAF-AID14 9J8h n=2
ubH2B ChIP
U2OS(FRT) Mock n=2
U2OS(FRT) 9J8h n=2
U2OS(FRT) CSB-KO Mock n=2
U2OS(FRT) CSB-KO 9J8h n=2
mean 100%
mean 63%
mean 98%
mean 38%
Normalized reads
Distance from TSS (bp)
-750 TSS
3000
TSS
100kb
ubH2B level (% of WT)

Supplementary Figure 6. All individual ChIP-seq replicates

Metaplots of all the individual PAF1 ChIP-seq, RNAPII ChIP-seq and Ub-H2B ChIP-seq replicates. PAF1 and RNAPII ChIP-seq plots present metaplots around the TSS of the top 3,000 genes of 3 to 100 kb in unirradiated and UV-irradiated U2OS WT, U2OS CSB-KO, U2OS TIR1, and U2OS PAF1-AID14 cells after normalization to area under the curve. Ub-H2B ChIP-seq plots present metaplots of 820 genes of >100 kb, where area under the curve was normalized to average Ub-H2B levels quantified by microscopy. Boxplots represent median, 5th and 95th percentile of this microscopy quantification in all cells of 2 independent experiments (see representative microscopy images in Figure 7j). The number of ChIP-seq replicates per condition are indicated in the plots.

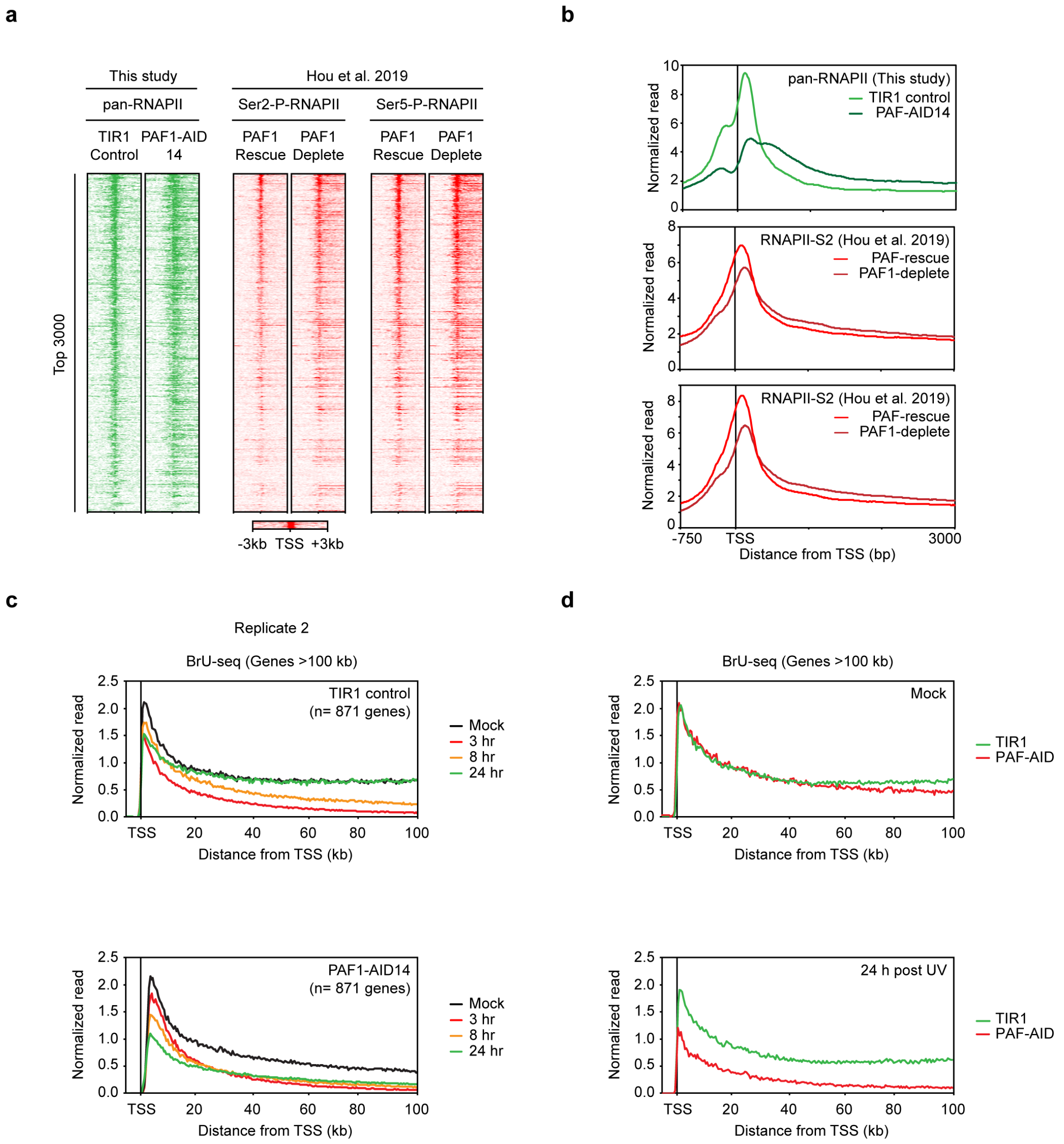
a
This study
pan-RNAPII
TIR1 Control
PAF1-AID 14
Hou et al. 2019
Ser2-P-RNAPII
Ser5-P-RNAPII
PAF1 Rescue
PAF1 Deplete
Top 3000
-3kb TSS +3kb
b
pan-RNAPII (This study)
TIR1 control
PAF-AID14
RNAPII-S2 (Hou et al. 2019)
PAF-rescue
PAF1-deplete
Normalized read
-750
TSS
3000
Distance from TSS (bp)
c
Replicate 2
BrU-seq (Genes >100 kb)
TIR1 control
(n= 871 genes)
PAF1-AID14
(n= 871 genes)
Mock
3 hr
8 hr
24 hr
Normalized read
Distance from TSS (kb)
d
BrU-seq (Genes >100 kb)
Mock
24 h post UV
TIR1
PAF-AID
Normalized read
Distance from TSS (kb)

Supplementary Figure 7. Comparison of ChIP-seq data in PAF1-depleted cells to published datasets and individual replicates of BrU-seq data

a Heatmaps around the TSS from our pan-RPB1 ChIP-seq data of the top 3,000 genes in auxin-treated U2OS TIR1 or PAF1-AID cells (This study; green). Data is compared to publish data (Hou *et al* 2019; red) presenting heatmaps around the same TSSs of Ser2-P-RNAPII or Ser5-P-RNAPII ChIP-seq data. **b** Averaged metaprofile presentations of the data in a. **c** Independent replicate of the BrU-seq experiment shown in Figure 8b. Metaplots of nascent transcription in genes of >100 kb in either TIR1 cells (top panel) or PAF1-AID cells (bottom panel) that were either mock-treated, or UV-irradiated (7 J/m^2) and analyzed at the indicated time-points (3, 8, or 24 hrs). The relative distribution of nascent transcript read density (in reads per thousand base-pairs per million reads) was normalized to the absolute nascent transcript intensities measured by 5-EU incorporation measurements that were performed in parallel to the BrU-seq experiments using the same cells and time-points (see Figure 4g, h). **d** Metaplots of BrU-seq reads densities (of replicate 1) comparing unirradiated (top panel) or UV-irradiated (bottom panel, 24 hours after UV) U2OS TIR1 cells to PAF-AID14 cells.

Supplementary Table 1. Cell lines used in this study

Cell line	Source
CS1AN-SV40 + GFP	LUMC, dr. L Mullenders
CS1AN-SV40 + GFP-CSB	LUMC, dr. L Mullenders
RPE1-hTERT(FRT)	Ximbio, London, UK
RPE1-hTERT(FRT) + GFP-LEO1 clone 6	This study
RPE1-hTERT(FRT) + GFP-NLS	This study
U2OS GFP-RPB1 clone I	LUMC, dr. H. van Attikum (*1*)
U2OS GFP-RPB1 CSA-KO clone 2-4	This study
U2OS GFP-RPB1 CSB-KO clone 1-40	This study
U2OS GFP-RPB1 UVSSA-KO clone 1-4	This study
U2OS TetOn-OsTIR1	LUMC, dr. H. van Attikum
U2OS TetOn-OsTIR1 PAF-AID clone 14	This study
U2OS TetOn-OsTIR1 PAF-AID clone 15	This study
U2OS(FRT)	This study
U2OS(FRT) + siRNA-resistant GFP-PAF1^{WT}	This study
U2OS(FRT) + siRNA-resistant GFP-PAF1$^{\Delta LEO1}$	This study
U2OS(FRT) CSA-KO + CSA-GFP (dox) clone 18	(*2*)
U2OS(FRT) CSA-KO clone 2-16	(*2*)
U2OS(FRT) CSB-KO + GFP-CSB (dox) clone 3	(*2*)
U2OS(FRT) CSB-KO clone 1-12	(*2*)
U2OS(FRT) UVSSA-KO + UVSSA-GFP (dox) clone 1-3	(*2*)
U2OS(FRT) UVSSA-KO clone 1-8	(*2*)
U2OS(FRT) XPA-KO + GFP-XPA (dox) clone 8	This study
U2OS(FRT) XPA-KO clone 2-8	This study
U2OS(FRT) XPC-KO clone 2-7	This study
XP168LV primary fibroblasts	Erasmus MC; dr. J. Marteijn (*3*)

Supplementary Table 2. sgRNAs used in this study

Target	Gene_ID	Exon_ID	Strand	Sequence
CSA	ENSG00000049167	ENSE00003589574	-1	CAACTTTGTGACTTGAAGTCTGG
CSB	ENSG00000225830	ENSE00002514316	-1	CCCCTCATCGGATCATTCTGTCT
UVSSA	ENSG00000163945	ENSE00001712443	1	AGAGAGCTGCTTTAGGCTGCTGG
XPA	ENSG00000136936	ENSE00003465148	-1	GCCCCAAAGATAATTGACACAGG
PAF1	ENSG00000006712	ENSE00001141439	-1	GAGTGACAGTGACTGAGTCCC

Supplementary Table 3. Primers used in this study

Sequencing primers for knockouts		
CSA	5-CATAGATAACTGAAATGTATGATTTCAAAG-3	oML#098_sgML#017_CSA_1_Fw
	5-CACAATCTTAGAGAAAGAAAGAATTTC-3	oML#099_sgML#017_CSA_1_Rv
	5-GTACATACATACATACACATTTACCAATAC-3	oML#100_sgML#018_CSA_2_Fw
	5-CTGAGAAAAAATGTACCTAAATATTAAG-3	oML#101_sgML#018_CSA_2_Rv
CSB	5-GTAGGGGCCAGTTGTTAGAATGTAA-3	oML#078_sgML#003_CSB1_Fw
	5-CTCACATTCTGAATGACTTGGCTA-3	oML#079_sgML#003_CSB1_Rv
UVSSA	5-ACCCAGAGGTACACAGAGATTG-3	oML#090_sgML#019_UVSSA1_Fw
	5-GCTCTTAGAAGTGTCCCTGTG-3	oML#091_sgML#019_UVSSA1_Rv
	5-ATCAGGAGGCTGAGGCGGCTG-3	oML#076_sgML#020_UVSSA2_Fw
	5-AGGAGCCTACCCGGGAGCCGGG-3	oML#077_sgML#020_UVSSA2_Rv
XPA	5-TTTTCAGGGCAGAGGTTGTA-3	oML#026 XPA-sgRNA2_Fw
	5-TCTTGGATTCTGGTGCCTCA-3	oML#027 XPA-sgRNA2_Rw
Primers for generating PAF-AID		
increasing multiple cloning site in pMK286 (clone XhoI x SacI)	5-ACAATTCTCGAGCAATTGGTCGACGGAT CCGGAGCTGGTGCAGGCGCCAAG-3	oML#155_AID-Fw
	5-GTGTAAGAGCTCCTGCAGCATATGAAG CTTATGGGTGGAGGCGGTTCATCAGAAG-3	oML#156_NEO-Rv
	5-GTGTAAGAGCTCCTGCAGCATATGAAGCT TATGGGTGGAGGCGGTTCATTAGCCCTC-3	oML#157_BLAST-Rv
PAF1 flank 1 (clone MfeI x BamHI)	5-ACAATTCAATTGCCGTATAACACACACACACAGTGTAGG-3	oML#158_PAF1 flank 1_MfeI_Fw
	5-GTGTAAGGATCCGACTCTGTCACTGTCACTATCAGC-3	oML#159_PAF1 flank 1_BamHI_Rv
PAF1 flank 2 (clone HindIII x SacI)	5-ACAATTAAGCTTCCAGGGCATTCAGGGCTGGTTC-3	oML#160_PAF1 flank 2 HindIII_Fw
	5-GTGTAAGAGCTCTTGCACACTCATCAACCTATGCAAG-3	oML#161_PAF1 flank 2_SacI_Rv
Primers for generating siPAF1 resistance		
siPAF1-2 resistance (clone EcoRV x AgeI)	5-GCACAAGCTGGAGTACAACTACAACAG-3	oML#011_GFP_clon_Fw
	5-GTATATTTCCTCTTCTGTGAATTGTTGTTTCACAG-3	oML#142_PAF siRNA-2resist-Rv
	5-CTGTGAAACAACAATTCACAGAAGAGGAAATATAC-3	oML#143_PAF siRNA-2resist-Fw
	5-CCTTGCTAGCTTTGTTCTTCACGTTC-3	oML#014_PAF_clon_Rv
siPAF1-3 resistance (clone EcoRV x NheI)	5-GCACAAGCTGGAGTACAACTACAACAG-3	oML#011_GFP_clon_Fw
	5-CAATTTTATAATCGTAGACGTCGTCTGGTG-3	oML#144_PAF siRNA-3resist-Rv
	5-CACCAGACGACGTCTACGATTATAAAATTG-3	oML#145_PAF siRNA-3resist-Fw
	5-GTGCCTAGCTTTGTTCTTCACGTTCCAG-3	oML#164_PAF1 NheI Rv
Primers for generating PAF1$^{\Delta LEO1}$		
(clone XmaI x NheI into AgeI x NheI)	5-ATTACCCGGGCTTCCCAGACTTTAAGATGTGGATC-3	oML#163_PAF1 XmaI-Fw (clone with AgeI)
	5-GTGCCTAGCTTTGTTCTTCACGTTCCAG-3	oML#164_PAF1 NheI Rv

Supplementary Table 4. siRNAs used in this study

Target	Sequence
Luciferase (Luc)	CGTACGCGGAATACTTCGA
PAF1-2	AAGCAGCAGTTTACCGAGGAA
PAF1-3	GATGATGTGTATGACTACA
XPA	CAGAGATGCTGATGATAAA
CSB-2	GAAGAGTTGTCAGTGATTA

Supplementary Table 5. Antibodies used in this study

Antibody	Host	Company (reference)	Use	Antibody identifier
A555 anti-mouse	Goat	Thermo Fisher (A-21424)	IF; 1:1,000	aML#015
Cas9	Mouse	Cell signalling (7A9-3A3 #14697)	WB: 1:2,000	aML#031
CF680 anti-rabbit	Goat	VWR (#20067)	WB; 1:10,000	aML#010
CF770 anti-mouse	Goat	VWR (#20077)	WB; 1:10,000	aML#009
CHD4	Rabbit	Active Motif (39289)	WB: 1:1,000	aML#019
CPD	Mouse	Cosmo Bio (CAC-NM-DND-001)	IF; 1:1000	aML#020
CSA	Rabbit	Abcam (ab137033)	WB: 1:750	aML#028
CSB	Rabbit	Santa Cruz (sc25370)	WB; 1:200	aML#003
CSB	Goat	Santa Cruz (SC-10459, E-18)	WB; 1:1,000	aML#039
CTR9	Rabbit	Bethyl (A301-395A)	WB; 1:5,000	aML#049
GFP	Mouse	Roche (11814460001)	WB; 1:1,000	aML#011
GFP	Rabbit	Abcam (ab290)	WB; 1:1,000	aML#044
LEO1	Rabbit	Bethyl, (A300-175A)	WB; 1:3,000	aML#023
PAF1	Rabbit	Bethyl (A300-172A)	WB; 1:3,000<br>ChIP; 3µg	aML#022
RPB1 (total)	Rabbit	Bethyl (A304-405A)	ChIP; 3µg	aML#088
RPB1-S2	Rabbit	Abcam (ab5095)	WB; 1:1,000	aML#024
RPB1-S5	Mouse	Abcam (ab5408)	WB: 1:1,000	-
Tubulin	Mouse	Sigma (T6199)	WB; 1:1,000	aML#008
ubH2B	Mouse	Abnova#10006	WB; 1:1,000	aML#059
ubH2B (K120)	Rabbit	Cell signaling (mAb#5546, D11)	ChIP; 3µg<br>IF; 1:200	-
XPA	Mouse	Invitrogen (MA5-13835)	WB; 1:500	aML#002
XPA	Rabbit	gift of Rick Wood (CJ1)	WB; 1:10,000	aML#079
XPC	Mouse	Abcam (ab6264)	WB: 1:500	aML#001

Supplementary Table 6. Sequence depth of individual ChIP-seq or BrU-seq replicates

	ChIP-antibody	Cells	Condition	Sample names individual replicates	Raw readcount	Unique, deduplicated and >q30
genomic DNA (ChIP)	PAF1; Bethyl (A300-172A)	U2OS WT	PAF1 WT mock (n=3)	data_PAF1_Total	41,414,021	33,493,159
				data_PAF1_2_Total	28,124,737	19,323,082
				data_PAF1_3_Total	22,806,181	17,767,146
			PAF1 WT 6J8h (n=1)	data_PAF16J_Total	21,122,168	15,082,669
			PAF1 WT 9J8h (n=3)	data_PAF19J_Total	5,081,473	3,359,766
				data_PAF19J_2_Total	31,269,025	21,181,598
				data_PAF19J_4_Total	16,581,452	12,841,951
			PAF1 WT 9J26h (n=1)	data_PAF19J26_1_Total	8,952,506	7,154,038
		U2OS CSB-KO	PAF1 CSB-KO mock (n=2)	data_PAF1CSBKO_UVneg_1_Total	36,097,216	27,403,853
				data_PAF1CSBKO_UVneg_3_Total	26,719,666	18,976,576
			PAF1 CSB-KO 6J8h (n=1)	data_PAF1CSBKO_6J_2_Total	18,172,814	14,503,974
			PAF1 CSB-KO 9J8h (n=2)	data_PAF1CSBKO_9J_1_Total	15,780,450	11,608,395
				data_PAF1CSBKO_9J_2_Total	17,683,210	13,503,020
	RPB1; Bethyl (A304-405A)	U2OS WT	RPB1 WT mock (n=3)	data_RNAPII_Total	41,395,370	33,433,766
				data_RNAPII_2_Total	31,770,432	23,849,268
				data_RNAPII_3_Total	35,355,782	27,176,383
			RPB1 WT 6J8h (n=3)	data_RNAPII6J_Total	44,551,507	36,205,677
				data_RNAPII6J_2_Total	39,096,246	28,920,544
				data_RNAPII6J_3_Total	32,543,776	20,662,551
			RPB1 WT 9J8h (n=2)	data_RNAPII9J_Total	11,688,852	8,792,714
				data_RNAPII9J_2_Total	28,144,751	17,504,545
			RPB1 WT 20J8h (n=1)	data_RNAPII20J_1_Total	24,148,823	16,276,244
			RPB1 WT 6J26h (n=2)	data_RNAPII6J26h_1_Total	9,742,407	7,786,496
				data_RNAPII6J26h_2_Total	24,798,918	20,104,022
			RPB1 WT 9J26h (n=2)	data_RNAPII9J26h_1_Total	42,198,702	34,648,355
				data_RNAPII9J26h_2_Total	19,935,049	15,732,660
		U2OS CSB-KO	RPB1 CSB-KO mock (n=2)	data_RNAPIICSBKO_UVneg_1_Total	38,725,120	30,045,175
				data_RNAPIICSBKO_UVneg_2_Total	22,758,682	16,994,571
			RPB1 CSB-KO 6J8h (n=2)	data_RNAPIICSBKO_6J_1_Total	42,020,519	27,716,826
				data_RNAPIICSBKO_6J_2_Total	21,486,820	15,617,139
		U2OS OsTIR1 control	OsTIR control mock (n=2)	data_TIR1_UVneg_1Combi_Total	39,632,412	27,383,117
				data_TIR1_UVneg_2_Total	23,482,185	18,735,016
			OsTIR control 6J8h (n=2)	data_TIR1_6J_1Combi_Total	49,035,212	37,911,176
				data_TIR1_6J_2_Total	19,375,328	15,549,197
			OsTIR control 9J8h (n=2)	data_TIR1_9J_1Combi_Total	41,773,346	32,468,368
				data_TIR1_9J_2_Total	19,515,592	15,737,017
		U2OS PAF-AID	PAF-AID mock (n=2)	data_PAFAID_UVneg_1Combi_Total	42,175,039	34,318,819
				data_PAFAID_UVneg_2_Total	23,690,651	19,392,119
			PAF-AID 6J8h (n=2)	data_PAFAID_6J_1Combi_Total	44,443,967	32,486,706
				data_PAFAID_6J_2_Total	18,520,546	14,744,881
			PAF-AID 9J8h (n=2)	data_PAFAID_9J_1Combi_Total	46,529,348	37,514,783
				data_PAFAID_9J_2_Total	19,723,345	15,912,500
	UbH2B (D11); Cell signalling (#5546)	U2OS WT	UbH2B WT mock (n=2)	WT_mock_rep1	56,520,876	43,563,596
				WT_mock_rep2	48,131,781	38,434,193
			UbH2B WT 9J8h (n=2)	WT_9J8h_rep1	50,120,670	35,016,326
				WT_9J8h_rep2	47,986,136	34,399,596
		U2OS CSB-KO	UbH2B CSBKO mock (n=2)	CSBKO_mock_rep1	42,558,206	31,764,080
				CSBKO_mock_rep2	50,593,317	39,024,693
			UbH2B CSBKO 9J8h (n=2)	CSBKO_9J8h_rep1	36,851,570	25,355,647
				CSBKO_9J8h_rep2	45,534,650	32,485,336
	not applicable	U2OS WT	Input WT mock (n=2)	data_Input5_Total (WT noUV)	23,152,371	18,929,239
				data_Input6_Total (WT noUV)	20,675,045	17,724,531
			Input WT 6J8h (n=1)	data_Input3_Total (WT 6J8h)	27,693,886	22,498,969
			Input WT 9J8h (n=1)	data_Input4_Total (WT 9J8h)	36,916,556	28,527,842
		U2OS CSB-KO	Input CSB-KO mock (n=1)	data_Input2_Total (CSB-KO noUV)	32,313,258	26,400,050
			Input CSB-KO 6J8h (n=1)	data_Input_Total (CSB-KO 6J8h)	28,124,309	23,271,565
			Input CSB-KO 9J8h (n=1)	CSBKO_9J8h_INPUT	44,243,247	24,750,537
		U2OS OsTIR1 control	Input OsTIR1 control mock (n=2)	data_InputOsTIR3_Total (noUV)	7,506,674	6,505,113
				data_InputOsTIR6_Total (noUV)	7,570,446	6,567,021
			Input OsTIR1 control 6J8h (n=2)	data_InputOsTIR_Total (6J8h)	6,439,103	5,608,462
				data_InputOsTIR4_Total (6J8h)	8,123,984	7,064,064
			Input OsTIR1 control 9J8h (n=2)	data_InputOsTIR2_Total (9J8h)	8,992,522	7,812,337
				data_InputOsTIR5_Total (9J8h)	6,910,620	6,003,073
nascent RNA (BrU-seq)	not applicable	U2OS OsTIR1 control	OsTIR1 mock (n=2)	TIR1_noUV_1	58,436,239	49,348,304
				TIR1_noUV_2	66,796,505	56,095,389
			OsTIR1 7J3h (n=2)	TIR1_3hUV_1	41,275,504	33,724,870
				TIR1_3hUV_2	65,786,084	49,872,648
			OsTIR1 7J8h (n=2)	TIR1_8hUV_1	50,255,096	42,518,104
				TIR1_8hUV_2	57,784,478	47,499,863

			OsTIR1 7J24h (n=2)	TIR1_24hUV_1	50,776,111	43,063,682
				TIR1_24hUV_2	88,533,628	73,845,426
		U2OS PAF-AID	PAF-AID mock (n=2)	PAFAID_noUV_1	40,177,346	29,454,444
				PAFAID_noUV_2	62,185,224	52,147,461
			PAF-AID 7J3h (n=2)	PAFAID_3hUV_1	47,350,367	35,015,351
				PAFAID_3hUV_2	58,910,610	43,061,294
			PAF-AID 7J8h (n=2)	PAFAID_8hUV_1	51,961,205	39,091,954
				PAFAID_8hUV_2	91,331,577	68,914,439
			PAF-AID 7J24h (n=2)	PAFAID_24hUV_1	46,432,487	23,803,165
				PAFAID_24hUV_2	113,928,601	88,962,601

References

1. P. Caron *et al.*, WWP2 ubiquitylates RNA polymerase II for DNA-PK-dependent transcription arrest and repair at DNA breaks. *Genes & development* **33**, 684-704 (2019).
2. Y. van der Weegen *et al.*, The cooperative action of CSB, CSA, and UVSSA target TFIIH to DNA damage-stalled RNA polymerase II. *Nat Commun* **11**, 2104 (2020).
3. F. Wienholz, W. Vermeulen, J. A. Marteijn, Amplification of unscheduled DNA synthesis signal enables fluorescence-based single cell quantification of transcription-coupled nucleotide excision repair. *Nucleic Acids Res* **45**, e68 (2017).
