# Supplementary material for: A CSB-PAF1C axis restores processive transcription elongation after DNA damage repair
Source: Nat Commun. 2021 Feb 26;12:1342. doi: 10.1038/s41467-021-21520-w (PMC7910549; doi:10.1038/s41467-021-21520-w)
Supplement: Supplementary file 3 — Reporting Summary [file 41467_2021_21520_MOESM3_ESM.pdf]

nature research

Corresponding author(s): M.S. Luijsterburg

Last updated by author(s): Jan 21, 2021

Reporting Summary

Nature Research wishes to improve the reproducibility of the work that we publish. This form provides structure for consistency and transparency in reporting. For further information on Nature Research policies, see Authors & Referees and the Editorial Policy Checklist.

Statistics

For all statistical analyses, confirm that the following items are present in the figure legend, table legend, main text, or Methods section.

n/a	Confirmed	
☐	☒	The exact sample size (*n*) for each experimental group/condition, given as a discrete number and unit of measurement
☐	☒	A statement on whether measurements were taken from distinct samples or whether the same sample was measured repeatedly
☐	☒	The statistical test(s) used AND whether they are one- or two-sided<br>*Only common tests should be described solely by name; describe more complex techniques in the Methods section.*
☒	☐	A description of all covariates tested
☒	☐	A description of any assumptions or corrections, such as tests of normality and adjustment for multiple comparisons
☐	☒	A full description of the statistical parameters including central tendency (e.g. means) or other basic estimates (e.g. regression coefficient) AND variation (e.g. standard deviation) or associated estimates of uncertainty (e.g. confidence intervals)
☐	☒	For null hypothesis testing, the test statistic (e.g. *F*, *t*, *r*) with confidence intervals, effect sizes, degrees of freedom and *P* value noted<br>*Give P values as exact values whenever suitable.*
☒	☐	For Bayesian analysis, information on the choice of priors and Markov chain Monte Carlo settings
☒	☐	For hierarchical and complex designs, identification of the appropriate level for tests and full reporting of outcomes
☒	☐	Estimates of effect sizes (e.g. Cohen's *d*, Pearson's *r*), indicating how they were calculated

Our web collection on statistics for biologists contains articles on many of the points above.

Software and code

Policy information about availability of computer code

Data collection

All software used in this study has been described in published literature and are detailed in the method section of the manuscript.
Software version:
MaxQuant (Version 1.5.3.30), Perseus Computational Platform (Version 1.5.5.3), ZEN 2012 (Blue edition, Version 1.1.0.0), Image J (Version1.48v), BWA (Version 0.7.16a), IGV (Version 2.4.3), MACS2 (Version 2.1), Samtools (Version 1.6), FastQC (Version 0.11.2), HOMER (Version 4.8.2), bowtie2 (Version 2.3.3.1), STAR (Version 2.7.0f), NCBI sratoolkit (Version 2.9.6-1-win64).
Further data analysis and presentation were performed with R (Version 3.5.3) and Rstudio (Version 1.1.423).

Data analysis

Mass spectrometry was analyzed using MaxQuant (Version 1.5.3.30) using standard settings with the following modifications. For the SILAC-labelled GFP-CSB samples, multiplicity was set to 2, marking Arg10 and Lys8 as heavy labels. Maximum missed cleavages by trypsin was set to 4. Searches were performed against an in silico digested database from the human proteome including isoforms and canonical proteins (Uniprot, 18th June 2018). Minimum peptide length was set to 6 aa and maximum peptide mass was set to 5 kDa. Carbamidomethyl (C) was disabled as fixed modification. The match between runs feature was activated. Minimum ratio count for quantification was set to 1. For the label-free GFP-LEO1 and GFP-RBP1 samples, maximum missed cleavages by trypsin was set to 4. Label-free quantification was activated, not enabling Fast LFQ. Searches were performed against an in silico digested database from the human proteome including isoforms and canonical proteins (Uniprot, 18th June 2018). Carbamidomethyl (C) was disabled as fixed modification. The match between runs feature was activated and iBAQ quantification was also enabled. MaxQuant output data from the SILAC samples analysis were further processed in Microsoft Excel 2016 for comprehensive visualization. Label-free analysis was further carried out in the Perseus Computational Platform (Version 1.5.5.3). LFQ intensity values were log2 transformed and potential contaminants and proteins identified by site only or reverse peptide were removed. Samples were grouped in experimental categories and proteins not identified in 4 out of 4 replicates in at least one group were also removed. Missing values were imputed using normally distributed values with a 1.8 downshift (log2) and a randomized 0.3 width (log2) considering whole matrix values.

Micropscopic mean intensities within the nucleus or local damage region was analyzed using ZEN 2012 (Blue edition, Version 1.1.0.0) and Image J (Version1.48v).

ChIP-seq and BrU-seq data analyses were performed using freely available tools, which are described in the methods section of the manuscript and in the data collection section above.
A first data quality check was performed using FastQC, considering read quality, level of duplications, GC-content, k-mer content and adapter contamination.
The BWA tool was used to align raw ChIP-seq reads to the Human Genome 38 (Hg38) (Li, 2013) and only unique and high-quality reads (> q30) were included.
MACS2 was used to generate bedgraph genome tracks and identify PAF1-binding peaks (Zhang et al., 2008), which were visualized in IGV (Version 2.4.3)
HOMER was used to convert bam files into TagDirectories and to define binding profiles (Heinz et al., 2010), using the settings described in the methods section of the manuscript.
R and Rstudio were further used to perform remaining customized analyses, using tools commonly available in R software. Custom code for these downstream analyses are available upon request.
NCBI sratoolkit was used to obtain published ChIP-seq datafiles from Epanchintsev and colleagues (Epanchintsev et al., 2017) (GSE87562), Hou and colleagues (Hou et al, 2019) (GSE116169) and Chen and colleagues (Chen et al., 2017) (GSE97527) and files were converted into FASTQ files and processed with the tools described above.
Bowtie2 was used to pre-filter BrU-seq reads by alignment to the human ribosomal repeating subunit (GenBank U13369.1) and human mitochondrial genome (chrM) from the hg38 reference genome.
STAR was used to map the remaining reads to the hg38 reference genome.
All detailed code including specified tool settings are available upon request to the corresponding author.

For manuscripts utilizing custom algorithms or software that are central to the research but not yet described in published literature, software must be made available to editors/reviewers. We strongly encourage code deposition in a community repository (e.g. GitHub). See the Nature Research guidelines for submitting code & software for further information.

Data

Policy information about availability of data

All manuscripts must include a data availability statement. This statement should provide the following information, where applicable:

- Accession codes, unique identifiers, or web links for publicly available datasets
- A list of figures that have associated raw data
- A description of any restrictions on data availability

Mass spectrometry proteomics data are presented in main Figs 1a, b, e, g, and Supplementary Figs S1d, h and S2f, and have been deposited to the ProteomeXchange Consortium via the PRIDE partner repository with the dataset identifier PXD016198 (https://www.ebi.ac.uk/pride/archive/projects/PXD016198). ChIP-seq data are presented in main Figs 5-7 and Supplementary Figs 4, 5, 6 and 7a, b. BrU-seq data are presented in mains Figure 8b-d and Supplementary Figs 7c, d. Both raw and processed ChIP-seq and BrU-seq data are deposited in the Gene Expression Omnibus under GSE140930 (https://www.ncbi.nlm.nih.gov/geo/query/acc.cgi?acc=GSE140930).
Additionally, previously published, publicly available, ChIP-seq datasets for RNAPII, ATF3, CSA, CSB and PAF1 (https://www.ncbi.nlm.nih.gov/geo/, GSE87562, GSE116169, GSE97527), and previously published protein structure data (https://www.rcsb.org/, 5VVR, 6GMH) has been obtained and used in this manuscript, as well as reference datasets of the Hg38 genome (https://ftp.ncbi.nlm.nih.gov/genomes/all/GCA/000/001/405/GCA_000001405.15_GRCh38/seqs_for_alignment_pipelines.ucsc_ids/GCA_000001405.15_GRCh38_no_alt_analysis_set.fna.gz), human ribosomal repeating subunit (GenBank U13369.1) and human mitochondrial genome (chrM) and the knownCanonical gene table from the UCSC genome database (https://genome.ucsc.edu/cgi-bin/hgTables, hg38 genome)

Source data are provided with this paper. Additional data will be made available upon reasonable request.

Field-specific reporting

Please select the one below that is the best fit for your research. If you are not sure, read the appropriate sections before making your selection.

☒ Life sciences ☐ Behavioural & social sciences ☐ Ecological, evolutionary & environmental sciences

For a reference copy of the document with all sections, see nature.com/documents/nr-reporting-summary-flat.pdf

Life sciences study design

All studies must disclose on these points even when the disclosure is negative.

Sample size	The number of replicate experiments are indicated in the figure legends of the manuscript and at least two replicates were performed for each individual approach, except when indicated otherwise. For some of the ChIP-seq experiments, replicates were defined as individual ChIP-sequencing experiments at different UV-C doses and/or different timepoints after UV treatment. Exact details on ChIP-seq replicates are indicated in Supplemental Table 1 and/ or in figure legends. A sample size of two was considered sufficient only when both repeats gave similar output and conclusions were confirmed using different methods. If not, at least three repeats were included in the analyses. A sample size of one was only included for experiments without major impact on conclusions and when results were confirmed by other methods. All replicates were included in plots presenting averages and representative examples were only presented when the majority of experiments confirmed that conclusion.
Data exclusions	Data were only excluded from the study when internal controls indicated evident technical errors such as a lack of proper locus enrichment in ChIP-seq.
Replication	- Mass spec was performed with 2-4 technical replicates and proteins were only included if they were observed in all replicates of the experiment. Findings were subsequently confirmed by reciprocal immunoprecipitation and western blot analyses<br>- ChIP-seq data in undamaged cells were compared to published datasets to confirm reliability of our ChIP-seq approach. In addition, ChIP-seq

was performed at different UV doses and time after UV to confirm reproducibility at varying damage load and recovery over time. The number of replicate experiments are indicated in the figure legends of the manuscript and at least two replicates were performed for each individual approach. For some of the ChIP-seq experiments, replicates were defined as individual ChIP-sequencing experiments at different UV-C doses and/or different timepoints after UV treatment. Exact details on ChIP-seq replicates are indicated in Supplemental Table 1 and/ or in figure legends. PAF1 ChIP-seq experiments were only rarely excluded due a lack of proper locus enrichment with the used PAF1 antibody. All ChIPs with a proper locus enrichment successfully confirmed our conclusions and are included in the manuscript.
- Effects of knock-down of proteins of interest were confirmed by using multiple independent siRNAs targeting the proteins of interest, and by re-expressing siRNA resistant constructs rescuing observed phenotypes. Knock-down efficiency or successful knock-out was confirmed by western blot prior to inclusion of downstream results.
- Successful co-immunoprecipitation was confirmed by detection of interactions described in literature. Representative co-immunoprecipitation experiments were only presented in the manuscript when the majority of the experiments confirmed the specific conclusion of the representative figure, which usually was the case for all successful co-immunoprecipitation replicates.

For the remaining experiments positive (often wild type) and negative (often NER deficient) control conditions were always included to verify reliability to the experiment. A sample size of two was considered sufficient only when both repeats gave similar output and conclusions were confirmed using different methods. If not, at least three repeats were included in the analyses. All replicates with confirmed controls were included in plots presenting averages, and representative examples were only presented when the majority of experiments confirmed that conclusion.

Randomization

Randomization was not applicable to our study since our study does not include patient or organism cohorts. We work with cell cultures and compare specific experimental conditions or wild type versus knock-out cell lines, which cannot be randomized

Blinding

Official blinding was not applicable to our study. Still for accuracy, microscopy was performed in an unbiased manner, selecting cells on DAPI signal without bias on output measures. Similarly, clonogenic survivals were quantified blinded to prevent subjective counting. Co-immunoprecipitation and ChIP-sequencing do not need blinding since all data is obtained and presented in an unbiased manner.

Reporting for specific materials, systems and methods

We require information from authors about some types of materials, experimental systems and methods used in many studies. Here, indicate whether each material, system or method listed is relevant to your study. If you are not sure if a list item applies to your research, read the appropriate section before selecting a response.

Materials & experimental systems

n/a	Involved in the study
☐	☒ Antibodies
☐	☒ Eukaryotic cell lines
☒	☐ Palaeontology
☒	☐ Animals and other organisms
☒	☐ Human research participants
☒	☐ Clinical data

Methods

n/a	Involved in the study
☐	☒ ChIP-seq
☒	☐ Flow cytometry
☒	☐ MRI-based neuroimaging

Antibodies

Antibodies used

A555 anti-mouse (Goat) - Thermo Fisher (A-21424); RRID:AB_141780
Cas9 (Mouse) - Cell signalling (7A9-3A3 #14697); RRID:AB_2750916; Lot#2
CF680 anti-rabbit (Goat) - VWR (#20067); RRID:AB_10871686
CF770 anti-mouse (Goat) - VWR (#20077); RRID: AB_10559194
CHD4 (Rabbit) - Active Motif (39289); RRID:AB_2614937; Lot# 01609002
CPD (Mouse) - Cosmo Bio (CAC-NM-DND-001); RRID:AB_1962813
CSA (Rabbit) - Abcam (ab137033); RRID:AB_2783825; Lot# GR155793-4
CSB (Rabbit) - Santa Cruz (sc25370); RRID:AB_668958; Lot#I1012
CSB (Goat) - Santa Cruz (SC-10459, E-18); RRID:AB_668957
CTR9 (Rabbit) - Bethyl (A301-395A); RRID:AB_960973; Lot#4
GFP (Mouse) - Roche (11814460001); RRID:AB_390913; Lot#27575600
GFP (Rabbit) - Abcam (ab290); RRID:AB_303395; Lot#GR3251545-1
LEO1 (Rabbit) - Bethyl, (A300-175A); RRID:AB_2135932; Lot#1
PAF1 (Rabbit) - Bethyl (A300-172A); RRID:AB_309394; Lot#2
RPB1-total (Rabbit) - Bethyl (A304-405A); RRID:AB_2620600; Lot#1
RPB1-S2 (Rabbit) - Abcam (ab5095); RRID:AB_304749; Lot#GR3231908-7
RPB1-S5 (Mouse) - Abcam (ab5408); RRID:AB_304868; Lot#GR3264297-4
Tubulin (Mouse) - Sigma (T6199); RRID:AB_477583; Lot#048M4751V
ubH2B (Mouse) - Abnova#10006; RRID:AB_10902597
ubH2B (K120) (Rabbit) - Cell signaling (mAb#5546, D11); RRID:AB_10693452
XPA (Mouse) - Invitrogen (MA5-13835); RRID:AB_10985162
XPA (Rabbit) - produced in the lab of Rick Wood (CJ1)
XPC (Mouse) - Abcam (ab6264); RRID:AB_305391

Validation

The following antibodies were validated in knockout cells: CSA (Rabbit) - Abcam (ab137033); RRID:AB_2783825; Lot# GR155793-4, CSB (Rabbit) - Santa Cruz (sc25370); RRID:AB_668958; Lot#I1012, CSB (Goat) - Santa Cruz (SC-10459, E-18);

RRID:AB_668957, XPA (Mouse) - Invitrogen (MA5-13835); RRID:AB_10985162, XPA (Rabbit) - produced in the lab of Rick Wood (CJ1), XPC (Mouse) - Abcam (ab6264); RRID:AB_305391.

The following antibodies were validated in Co-IP experiments:
CTR9 (Rabbit) - Bethyl (A301-395A); RRID:AB_960973; Lot#4, LEO1 (Rabbit) - Bethyl, (A300-175A); RRID:AB_2135932; Lot#1, PAF1 (Rabbit) - Bethyl (A300-172A); RRID:AB_309394; Lot#2, RPB1-S2 (Rabbit) - Abcam (ab5095); RRID:AB_304749; Lot#GR3231908-7, RPB1-S5 (Mouse) - Abcam (ab5408); RRID:AB_304868; Lot#GR3264297-4.

This antibody was validated by an in-frame endogenous knock-in of an auxin-inducible degron sequence causing a size change in the targeted protein:
PAF1 (Rabbit) - Bethyl (A300-172A); RRID:AB_309394; Lot#2.

The following antibodies were validated in knock-down experiments:
CHD4 (Rabbit) - Active Motif (39289); RRID:AB_2614937; Lot# 01609002, PAF1 (Rabbit) - Bethyl (A300-172A); RRID:AB_309394; Lot#2.

The following antibodies were validated by over-expression experiments:
Cas9 (Mouse) - Cell signalling (7A9-3A3 #14697); RRID:AB_2750916; Lot#2, GFP (Mouse) - Roche (11814460001); RRID:AB_390913; Lot#27575600, GFP (Rabbit) - Abcam (ab290); RRID:AB_303395; Lot#GR3251545-1.

These antibodies were validated in ChIP-seq experiments:
RPB1-total (Rabbit) - Bethyl (A304-405A); RRID:AB_2620600; Lot#1, ubH2B (K120) (Rabbit) - Cell signaling (mAb#5546, D11); RRID:AB_10693452

This antibody is a commonly used loading control:
Tubulin (Mouse) - Sigma (T6199); RRID:AB_477583; Lot#048M4751V

Eukaryotic cell lines

Policy information about cell lines

Cell line source(s)

CS1AN-SV40 + GFP (LUMC, dr. L Mullenders)
CS1AN-SV40 + GFP-CSB (LUMC, dr. L Mullenders)
RPE1-hTERT-Flp-In/T-Rex (RPE-hTERT(FRT)) (Ximbio, London, UK)
RPE-hTERT(FRT) + GFP-LEO1 clone 6 (This study)
RPE-hTERT(FRT) + GFP-NLS (This study)
U2OS GFP-RPB1 clone I (LUMC, dr. H. van Attikum; Caron et al., 2019)
U2OS GFP-RPB1 CSA-KO clone 2-4 (This study)
U2OS GFP-RPB1 CSB-KO clone 1-40 (This study)
U2OS GFP-RPB1 UVSSA-KO clone 1-4 (This study)
U2OS TetOn-OsTIR1 (LUMC, dr. H. van Attikum)
U2OS TetOn-OsTIR1 PAF-AID clone 14 (This study)
U2OS TetOn-OsTIR1 PAF-AID clone 15 (This study)
U2OS-Flp-In/T-Rex (U2OS(FRT)) (LUMC, Van der Weegen; Van der Weegen et al., 2020)
U2OS(FRT) + siRNA-resistant GFP-PAF1(WT) (This study)
U2OS(FRT) + siRNA-resistant GFP-PAF1(ΔLEO1) (This study)
U2OS(FRT) CSA-KO + CSA-GFP (dox) clone 18 (This study)
U2OS(FRT) CSA-KO clone 2-16 (This study)
U2OS(FRT) CSB-KO + GFP-CSB (dox) clone 3 (LUMC, Van der Weegen; Van der Weegen et al., 2020)
U2OS(FRT) CSB-KO clone 1-12 (LUMC, Van der Weegen; Van der Weegen et al., 2020)
U2OS(FRT) UVSSA-KO + UVSSA-GFP (dox) clone 1-3 (LUMC, Van der Weegen; Van der Weegen et al., 2020)
U2OS(FRT) UVSSA-KO clone 1-8 (LUMC, Van der Weegen; Van der Weegen et al., 2020)
U2OS(FRT) XPA-KO + GFP-XPA (dox) clone 8 (This study)
U2OS(FRT) XPA-KO clone 2-8 (LUMC, Van der Weegen; Van der Weegen et al., 2020)
U2OS(FRT) XPC-KO clone 2-7 (LUMC, Van der Weegen; Van der Weegen et al., 2020)
XP168LV primary fibroblasts (Erasmus MC; dr. J. Marteijn; Wienholz et al., 2017)

Authentication

Cells were authenticated by STR profiling. All knockout cells were validated by Western blot analysis and DNA sequencing

Mycoplasma contamination

All cell lines were routinely tested for mycoplasma and were nested negative

Commonly misidentified lines
(See ICLAC register)

No commonly misidentified cell lines were used in this study

ChIP-seq

Data deposition

☒ Confirm that both raw and final processed data have been deposited in a public database such as GEO.

☒ Confirm that you have deposited or provided access to graph files (e.g. BED files) for the called peaks.

Data access links
May remain private before publication.

Both raw and processed ChIP-seq and BrU-seq data are deposited in the Gene Expression Omnibus (GEO; https://www.ncbi.nlm.nih.gov/geo/) using the identifier GSE140930

Additional data will be provided upon request

Files in database submission

Raw data
ChIP-seq: Inputs
CSBKO_UV_INPUT_1.fastq.gz
CSBKO_UV_INPUT_2.fastq.gz
PAF1-RPB-20180926-input-CSB-KO-6J8hUV-16178_R1.fastq.gz
PAF1-RPB-20180926-input-CSB-KO-6J8hUV-16178_R2.fastq.gz
PAF1-RPB-20180926-input-CSB-KO-noUV-16177_R1.fastq.gz
PAF1-RPB-20180926-input-CSB-KO-noUV-16177_R2.fastq.gz
PAF1-RPB-20180926-input-WT-6J8hUV-16175_R1.fastq.gz
PAF1-RPB-20180926-input-WT-6J8hUV-16175_R2.fastq.gz
PAF1-RPB-20180926-input-WT-9J8hUV-16176_R1.fastq.gz
PAF1-RPB-20180926-input-WT-9J8hUV-16176_R2.fastq.gz
PAF1-RPB-20180926-input-WT-noUV-16174_R1.fastq.gz
PAF1-RPB-20180926-input-WT-noUV-16174_R2.fastq.gz
U2OS-input-13462_R1.fastq.gz
U2OS-input-13462_R2.fastq.gz
PAF1-RPB-20190123-INPUT-OsTIRcontrol-6J8h-14-16502_R1.fastq.gz
PAF1-RPB-20190123-INPUT-OsTIRcontrol-6J8h-14-16502_R2.fastq.gz
PAF1-RPB-20190123-INPUT-OsTIRcontrol-9J8h-15-16503_R1.fastq.gz
PAF1-RPB-20190123-INPUT-OsTIRcontrol-9J8h-15-16503_R2.fastq.gz
PAF1-RPB-20190123-INPUT-OsTIRcontrol-noUV-13-16501_R1.fastq.gz
PAF1-RPB-20190123-INPUT-OsTIRcontrol-noUV-13-16501_R2.fastq.gz
PAF1-RPB-20190123-INPUT-PAFAID14-6J8h-17-16505_R1.fastq.gz
PAF1-RPB-20190123-INPUT-PAFAID14-6J8h-17-16505_R2.fastq.gz
PAF1-RPB-20190123-INPUT-PAFAID14-9J8h-18-16506_R1.fastq.gz
PAF1-RPB-20190123-INPUT-PAFAID14-9J8h-18-16506_R2.fastq.gz
PAF1-RPB-20190123-INPUT-PAFAID14-noUV-16-16504_R1.fastq.gz
PAF1-RPB-20190123-INPUT-PAFAID14-noUV-16-16504_R2.fastq.gz

ChIP-seq: antibody – PAF1; Bethyl (A300-172A)
PAF1-RPB-20180724-PAF1-WT-noUV-15770_R1.fastq.gz
PAF1-RPB-20180724-PAF1-WT-noUV-15770_R2.fastq.gz
PAF1-RPB-20180926-PAF1-WT-noUV-16179_R1.fastq.gz
PAF1-RPB-20180926-PAF1-WT-noUV-16179_R2.fastq.gz
PAF1-WT-U2OS-13456_R1.fastq.gz
PAF1-WT-U2OS-13456_R2.fastq.gz
PAF1-RPB-20180926-PAF1-WT-6J8hUV-16180_R1.fastq.gz
PAF1-RPB-20180926-PAF1-WT-6J8hUV-16180_R2.fastq.gz
PAF1-RPB-20180926-PAF1-WT-9J8hUV-16181_R1.fastq.gz
PAF1-RPB-20180926-PAF1-WT-9J8hUV-16181_R2.fastq.gz
PAF1-RPB-20190220-PAF1-WT-9J8h-rep4-16660_R1.fastq.gz
PAF1-RPB-20190220-PAF1-WT-9J8h-rep4-16660_R2.fastq.gz
PAF1-RPB-20180424-PAF1-9J8hUV-U2OS-15539_R1.fastq.gz
PAF1-RPB-20180424-PAF1-9J8hUV-U2OS-15539_R2.fastq.gz
PAF1-RPB-20180424-PAF1-9J26hUV-U2OS-15540_R1.fastq.gz
PAF1-RPB-20180424-PAF1-9J26hUV-U2OS-15540_R2.fastq.gz
PAF1-RPB-20180926-PAF1-CSBKO-6J8h-spikein-16653_R1.fastq.gz
PAF1-RPB-20180926-PAF1-CSBKO-6J8h-spikein-16653_R2.fastq.gz
PAF1-RPB-20190220-PAF1-CSBKO-9J8h-rep1-16661_R1.fastq.gz
PAF1-RPB-20190220-PAF1-CSBKO-9J8h-rep1-16661_R2.fastq.gz
PAF1-RPB-20190220-PAF1-CSBKO-9J8h-rep2-16662_R1.fastq.gz
PAF1-RPB-20190220-PAF1-CSBKO-9J8h-rep2-16662_R2.fastq.gz
PAF1-RPB-20180926-PAF1-CSBKO-noUV-spikein-16652_R1.fastq.gz
PAF1-RPB-20180926-PAF1-CSBKO-noUV-spikein-16652_R2.fastq.gz
PAF1-KO-U2OS-13457_R1.fastq.gz
PAF1-KO-U2OS-13457_R2.fastq.gz

ChIP-seq: antibody – RPB1; Bethyl (A304-405A)
PAF1-RPB-20180724-RPB1-WT-noUV-15772_R1.fastq.gz
PAF1-RPB-20180724-RPB1-WT-noUV-15772_R2.fastq.gz
PAF1-RPB-20180926-RPB1-WT-noUV-16182_R1.fastq.gz
PAF1-RPB-20180926-RPB1-WT-noUV-16182_R2.fastq.gz
PAF1-RPB-20180424-RPB1-noUV-U2OS-15541_R1.fastq.gz
PAF1-RPB-20180424-RPB1-noUV-U2OS-15541_R2.fastq.gz
PAF1-RPB-20180724-RPB1-WT-20J8h-15773_R1.fastq.gz
PAF1-RPB-20180724-RPB1-WT-20J8h-15773_R2.fastq.gz
PAF1-RPB-20180724-RPB1-WT-6J8h-15774_R1.fastq.gz
PAF1-RPB-20180724-RPB1-WT-6J8h-15774_R2.fastq.gz
PAF1-RPB-20180926-RPB1-WT-6J8hUV-16183_R1.fastq.gz
PAF1-RPB-20180926-RPB1-WT-6J8hUV-16183_R2.fastq.gz
PAF1-RPB-20180424-RPB1-6J8hUV-U2OS-15542_R1.fastq.gz
PAF1-RPB-20180424-RPB1-6J8hUV-U2OS-15542_R2.fastq.gz

PAF1-RPB-20180424-RPB1-6J26hUV-U2OS-15543_R1.fastq.gz
PAF1-RPB-20180424-RPB1-6J26hUV-U2OS-15543_R2.fastq.gz
PAF1-RPB-20190123-RPB1-WT-6J26h-1-16489_R1.fastq.gz
PAF1-RPB-20190123-RPB1-WT-6J26h-1-16489_R2.fastq.gz
PAF1-RPB-20180926-RPB1-WT-9J8hUV-16184_R1.fastq.gz
PAF1-RPB-20180926-RPB1-WT-9J8hUV-16184_R2.fastq.gz
PAF1-RPB-20180424-RPB1-9J8hUV-U2OS-15544_R1.fastq.gz
PAF1-RPB-20180424-RPB1-9J8hUV-U2OS-15544_R2.fastq.gz
PAF1-RPB-20180424-RPB1-9J26hUV-U2OS-15545_R1.fastq.gz
PAF1-RPB-20180424-RPB1-9J26hUV-U2OS-15545_R2.fastq.gz
PAF1-RPB-20190123-RPB1-WT-9J26h-2-16490_R1.fastq.gz
PAF1-RPB-20190123-RPB1-WT-9J26h-2-16490_R2.fastq.gz
PAF1-RPB-20180724-RPB1-CSB-KO-6J8h-15777_R1.fastq.gz
PAF1-RPB-20180724-RPB1-CSB-KO-6J8h-15777_R2.fastq.gz
PAF1-RPB-20180926-RPB1-CSB-KO-6J8hUV-16186_R1.fastq.gz
PAF1-RPB-20180926-RPB1-CSB-KO-6J8hUV-16186_R2.fastq.gz
PAF1-RPB-20180724-RPB1-CSB-KO-noUV-15775_R1.fastq.gz
PAF1-RPB-20180724-RPB1-CSB-KO-noUV-15775_R2.fastq.gz
PAF1-RPB-20180926-RPB1-CSB-KO-noUV-16185_R1.fastq.gz
PAF1-RPB-20180926-RPB1-CSB-KO-noUV-16185_R2.fastq.gz
PAF1-RPB-20190123-RPB1-OsTIRcontrol-6J8h-4-16492_Combined_R1.fastq.gz
PAF1-RPB-20190123-RPB1-OsTIRcontrol-6J8h-4-16492_Combined_R2.fastq.gz
PAF1-RPB-20190220-RPB1-OsTIR1control-6J8h-rep2-16655_R1.fastq.gz
PAF1-RPB-20190220-RPB1-OsTIR1control-6J8h-rep2-16655_R2.fastq.gz
PAF1-RPB-20190123-RPB1-OsTIRcontrol-9J8h-5-16493_Combined_R1.fastq.gz
PAF1-RPB-20190123-RPB1-OsTIRcontrol-9J8h-5-16493_Combined_R2.fastq.gz
PAF1-RPB-20190220-RPB1-OsTIR1control-9J8h-rep2-16656_R1.fastq.gz
PAF1-RPB-20190220-RPB1-OsTIR1control-9J8h-rep2-16656_R2.fastq.gz
PAF1-RPB-20190123-RPB1-OsTIRcontrol-noUV-3-16491_Combined_R1.fastq.gz
PAF1-RPB-20190123-RPB1-OsTIRcontrol-noUV-3-16491_Combined_R2.fastq.gz
PAF1-RPB-20190220-RPB1-OsTIR1control-noUV-rep2-16654_R1.fastq.gz
PAF1-RPB-20190220-RPB1-OsTIR1control-noUV-rep2-16654_R2.fastq.gz
PAF1-RPB-20190123-RPB1-PAFAID14-6J8h-7-16495_Combined_R1.fastq.gz
PAF1-RPB-20190123-RPB1-PAFAID14-6J8h-7-16495_Combined_R2.fastq.gz
PAF1-RPB-20190220-RPB1-PAFAID14-6J8h-rep2-16658_R1.fastq.gz
PAF1-RPB-20190220-RPB1-PAFAID14-6J8h-rep2-16658_R2.fastq.gz
PAF1-RPB-20190123-RPB1-PAFAID14-9J8h-8-16496_Combined_R1.fastq.gz
PAF1-RPB-20190123-RPB1-PAFAID14-9J8h-8-16496_Combined_R2.fastq.gz
PAF1-RPB-20190220-RPB1-PAFAID14-9J8h-rep2-16659_R1.fastq.gz
PAF1-RPB-20190220-RPB1-PAFAID14-9J8h-rep2-16659_R2.fastq.gz
PAF1-RPB-20190123-RPB1-PAFAID14-noUV-6-16494_Combined_R1.fastq.gz
PAF1-RPB-20190123-RPB1-PAFAID14-noUV-6-16494_Combined_R2.fastq.gz
PAF1-RPB-20190220-RPB1-PAFAID14-noUV-rep2-16657_R1.fastq.gz
PAF1-RPB-20190220-RPB1-PAFAID14-noUV-rep2-16657_R2.fastq.gz

ChIP-seq: antibody – ubH2B (K120) - Cell signaling (mAb#5546, D11)
CSB_mock_rep1_1.fastq.gz
CSB_mock_rep1_2.fastq.gz
CSB_mock_rep2_1.fastq.gz
CSB_mock_rep2_2.fastq.gz
CSB_UV_rep1_1.fastq.gz
CSB_UV_rep1_2.fastq.gz
CSB_UV_rep2_1.fastq.gz
CSB_UV_rep2_2.fastq.gz
WT_mock_rep1_1.fastq.gz
WT_mock_rep1_2.fastq.gz
WT_mock_rep2_1.fastq.gz
WT_mock_rep2_2.fastq.gz
WT_UV_rep1_1.fastq.gz
WT_UV_rep1_2.fastq.gz
WT_UV_rep2_1.fastq.gz
WT_UV_rep2_2.fastq.gz

BrU-seq:
TIR1_noUV_1 – 138119_trimmed_S_R1_001.fastq.gz
TIR1_3hUV_1 – 138120_trimmed_S_R1_001.fastq.gz
TIR1_8hUV_1 – 138121_trimmed_S_R1_001.fastq.gz
TIR1_24hUV_1 – 138122_trimmed_S_R1_001.fastq.gz
TIR1_noUV_2 – 140500_trimmed_S_R1_001.fastq.gz
TIR1_3hUV_2 – 140501_trimmed_S_R1_001.fastq.gz
TIR1_8hUV_2 – 140502_trimmed_S_R1_001.fastq.gz
TIR1_24hUV_2 – 140503_trimmed_S_R1_001.fastq.gz
PAFAID_noUV_1 – 138123_trimmed_S_R1_001.fastq.gz
PAFAID_3hUV_1 – 138124_trimmed_S_R1_001.fastq.gz
PAFAID_8hUV_1 – 138125_trimmed_S_R1_001.fastq.gz

PAFAID_24hUV_1 – 138126_trimmed_S_R1_001.fastq.gz
PAFAID_noUV_2 – 140504_trimmed_S_R1_001.fastq.gz
PAFAID_3hUV_2 – 140505_trimmed_S_R1_001.fastq.gz
PAFAID_8hUV_2 – 140506_trimmed_S_R1_001.fastq.gz
PAFAID_24hUV_2 – 140507_trimmed_S_R1_001.fastq.gz

Processed data (additional data will be made available upon request)
ChIP-seq:
averages_Normalized_750TSS3000_Top3000.txt
RNAPII_TravelingRatio_Top3000.txt
UbH2B_Distribution_Above100kbGenes_averages.xlsx

BrU-seq:
BrUseq_Average.xlsx

Genome browser session
(e.g. UCSC)

ChIP-seq and BrU-seq example tracks have been generated from Bedgraph/BigWig files using IGV (Version 2.4.3) and not using a UCSC genome browser session

Methodology

Replicates

ChIP-seq data in undamaged cells were compared to published datasets to confirm reliability of our ChIP-seq approach. In addition, ChIP-seq was performed at different UV doses to confirm reproducibility at varying damage loads and at different time-points after UV. The number of replicates are indicated in Supplemental Table 6 and related figure legends.

Sequencing depth

All ChIP-seq samples were sequenced in paired-end with 42nt or 151nt sequencing reads. All BrU-seq samples were sequenced in paired-end (although used single-end) with 151nt sequencing reads. Details on sequencing depth and uniquely mapped high quality reads are described in Supplemental Table 6 and added below.

ChIP-seq
PAF1 WT mock (n=3)
data_PAF1_Total Total: 41,414,021 Unique >q30: 33,493,159
data_PAF1_2_Total Total: 28,124,737 Unique >q30: 19,323,082
data_PAF1_3_Total Total: 22,806,181 Unique >q30: 17,767,146

PAF1 WT 6J8h (n=1)
data_PAF16J_Total Total: 21,122,168 Unique >q30: 15,082,669

PAF1 WT 9J8h (n=3)
data_PAF19J_Total Total: 5,081,473 Unique >q30: 3,359,766
data_PAF19J_2_Total Total: 31,269,025 Unique >q30: 21,181,598
data_PAF19J_4_Total Total: 16,581,452 Unique >q30: 12,841,951

PAF1 WT 9J26h (n=1)
data_PAF19J26_1_Total Total: 8,952,506 Unique >q30: 7,154,038

PAF1 CSB-KO 6J8h (n=1)
data_PAF1CSBKO_6J_2_Total Total: 18,172,814 Unique >q30: 14,503,974

PAF1 CSB-KO 9J8h (n=2)
data_PAF1CSBKO_9J_1_Total Total: 15,780,450 Unique >q30: 11,608,395
data_PAF1CSBKO_9J_2_Total Total: 17,683,210 Unique >q30: 13,503,020

PAF1 CSB-KO mock (n=2)
data_PAF1CSBKO_UVneg_1_Total Total: 36,097,216 Unique >q30: 27,403,853
data_PAF1CSBKO_UVneg_3_Total Total: 26,719,666 Unique >q30: 18,976,576

RPB1 WT mock (n=3)
data_RNAPII_Total Total: 41,395,370 Unique >q30: 33,433,766
data_RNAPII_2_Total Total: 31,770,432 Unique >q30: 23,849,268
data_RNAPII_3_Total Total: 35,355,782 Unique >q30: 27,176,383

RPB1 WT 6J8h (n=3)
data_RNAPII6J_Total Total: 44,551,507 Unique >q30: 36,205,677
data_RNAPII6J_2_Total Total: 39,096,246 Unique >q30: 28,920,544
data_RNAPII6J_3_Total Total: 32,543,776 Unique >q30: 20,662,551

RPB1 WT 9J8h (n=2)
data_RNAPII9J_Total Total: 11,688,852 Unique >q30: 8,792,714
data_RNAPII9J_2_Total Total: 28,144,751 Unique >q30: 17,504,545

RPB1 WT 20J8h (n=1)
data_RNAPII20J_1_Total Total: 24,148,823 Unique >q30: 16,276,244

RPB1 WT 6J26h (n=2)

data_RNAPII6J26h_1_Total Total: 9,742,407 Unique >q30: 7,786,496
data_RNAPII6J26h_2_Total Total: 24,798,918 Unique >q30: 20,104,022

RPB1 WT 9J26h (n=2)
data_RNAPII9J26h_1_Total Total: 42,198,702 Unique >q30: 34,648,355
data_RNAPII9J26h_2_Total Total: 19,935,049 Unique >q30: 15,732,660

RPB1 CSB-KO mock (n=2)
data_RNAPIICSBKO_UVneg_1_Total Total: 38,725,120 Unique >q30: 30,045,175
data_RNAPIICSBKO_UVneg_2_Total Total: 22,758,682 Unique >q30: 16,994,571

RPB1 CSB-KO 6J8h (n=2)
data_RNAPIICSBKO_6J_1_Total Total: 42,020,519 Unique >q30: 27,716,826
data_RNAPIICSBKO_6J_2_Total Total: 21,486,820 Unique >q30: 15,617,139

RPB1 OsTIR control mock (n=2)
data_TIR1_UVneg_1Combi_Total Total: 39,632,412 Unique >q30: 27,383,117
data_TIR1_UVneg_2_Total Total: 23,482,185 Unique >q30: 18,735,016

RPB1 OsTIR control 6J8h (n=2)
data_TIR1_6J_1Combi_Total Total: 49,035,212 Unique >q30: 37,911,176
data_TIR1_6J_2_Total Total: 19,375,328 Unique >q30: 15,549,197

RPB1 OsTIR control 9J8h (n=2)
data_TIR1_9J_1Combi_Total Total: 41,773,346 Unique >q30: 32,468,368
data_TIR1_9J_2_Total   Total: 19,515,592 Unique >q30: 15,737,017

RPB1 PAF-AID mock (n=2)
data_PAFAID_UVneg_1Combi_Total Total: 42,175,039 Unique >q30: 34,318,819
data_PAFAID_UVneg_2_Total Total: 23,690,651 Unique >q30: 19,392,119

RPB1 PAF-AID 6J8h (n=2)
data_PAFAID_6J_1Combi_Total Total: 44,443,967 Unique >q30: 32,486,706
data_PAFAID_6J_2_Total Total: 18,520,546 Unique >q30: 14,744,881

RPB1 PAF-AID 9J8h (n=2)
data_PAFAID_9J_1Combi_Total Total: 46,529,348 Unique >q30: 37,514,783
data_PAFAID_9J_2_Total Total: 19,723,345 Unique >q30: 15,912,500

Ubi-H2B WT mock (n=2)
WT_mock_rep1 Total: 56,520,876 Unique >q30: 43,563,596
WT_mock_rep2 Total: 48,131,781 Unique >q30: 38,434,193

Ubi-H2B WT 9J8h (n=2)
WT_9J8h_rep1 Total: 50,120,670 Unique >q30: 35,016,326
WT_9J8h_rep2 Total: 47,986,136 Unique >q30: 34,399,596

Ubi-H2B CSBKO mock (n=2)
CSBKO_mock_rep1 Total: 42,558,206 Unique >q30: 31,764,080
CSBKO_mock_rep2 Total: 50,593,317 Unique >q30: 39,024,693

Ubi-H2B CSBKO 9J8h (n=2)
CSBKO_9J8h_rep1 Total: 36,851,570 Unique >q30: 25,355,647
CSBKO_9J8h_rep2 Total: 45,534,650 Unique >q30: 32,485,336

Input WT mock (n=2)
data_Input5_Total (WT noUV) Total: 23,152,371 Unique >q30: 18,929,239
data_Input6_Total (WT noUV) Total: 20,675,045 Unique >q30: 17,724,531

Input WT 6J8h (n=1)
data_Input3_Total (WT 6J8h) Total: 27,693,886 Unique >q30: 22,498,969

Input WT 9J8h (n=1)
data_Input4_Total (WT 9J8h) Total: 36,916,556 Unique >q30: 28,527,842

Input CSB-KO mock (n=1)
data_Input2_Total (CSB-KO noUV) Total: 32,313,258 Unique >q30: 26,400,050

Input CSB-KO 6J8h (n=1)
data_Input_Total (CSB-KO 6J8h) Total: 28,124,309 Unique >q30: 23,271,565

Input CSB-KO 9J8h (n=1)
CSBKO_9J8h_INPUT Total: 44,243,247 Unique >q30: 24,750,537

Input OsTIR1 control mock (n=2)
data_InputOsTIR3_Total (noUV) Total: 7,506,674 Unique >q30: 6,505,113
data_InputOsTIR6_Total (noUV) Total: 7,570,446 Unique >q30: 6,567,021

Input OsTIR1 control 6J8h (n=2)
data_InputOsTIR_Total (6J8h) Total: 6,439,103 Unique >q30: 5,608,462
data_InputOsTIR4_Total (6J8h) Total: 8,123,984 Unique >q30: 7,064,064

Input OsTIR1 control 9J8h (n=2)
data_InputOsTIR2_Total (9J8h) Total: 8,992,522 Unique >q30: 7,812,337
data_InputOsTIR5_Total (9J8h) Total: 6,910,620 Unique >q30: 6,003,073

BrU-seq

OsTIR1 mock (n=2)
TIR1_noUV_1 Total: 58,436,239 Unique >q30: 49,348,304
TIR1_noUV_2 Total: 66,796,505 Unique >q30: 56,095,389

OsTIR1 7J3h (n=2)
TIR1_3hUV_1 Total: 41,275,504 Unique >q30: 33,724,870
TIR1_3hUV_2 Total: 65,786,084 Unique >q30: 49,872,648

OsTIR1 7J8h (n=2)
TIR1_8hUV_1 Total: 50,255,096 Unique >q30: 42,518,104
TIR1_8hUV_2 Total: 57,784,478 Unique >q30: 47,499,863

OsTIR1 7J24h (n=2)
TIR1_24hUV_1 Total: 50,776,111 Unique >q30: 43,063,682
TIR1_24hUV_2 Total: 88,533,628 Unique >q30: 73,845,426

PAF-AID mock (n=2)
PAFAID_noUV_1 Total: 40,177,346 Unique >q30: 29,454,444
PAFAID_noUV_2 Total: 62,185,224 Unique >q30: 52,147,461

PAF-AID 7J3h (n=2)
PAFAID_3hUV_1 Total: 47,350,367 Unique >q30: 35,015,351
PAFAID_3hUV_2 Total: 58,910,610 Unique >q30: 43,061,294

PAF-AID 7J8h (n=2)
PAFAID_8hUV_1 Total: 51,961,205 Unique >q30: 39,091,954
PAFAID_8hUV_2 Total: 91,331,577 Unique >q30: 68,914,439

PAF-AID 7J24h (n=2)
PAFAID_24hUV_1 Total: 46,432,487 Unique >q30: 23,803,165
PAFAID_24hUV_2 Total: 113,928,601 Unique >q30: 88,962,601

Antibodies

RPB1-total (Rabbit) - Bethyl (A304-405A); RRID:AB_2620600; Lot#1
PAF1 (Rabbit) - Bethyl (A300-172A); RRID:AB_309394; Lot#2
ubH2B (K120) (Rabbit) - Cell signaling (mAb#5546, D11); RRID:AB_10693452
For all antibodies we used 3µg antibody per ChIP-seq experiment

Peak calling parameters

Peak calling was performed using the freely available MACS2 software with PAF1 ChIP-seq data in wild-type undamaged cells or UV-irradiated cell relative to a matched UV-negative or UV-treated input sample.

Data quality

Data quality for individual replicates was confirmed by FastQC checking ead quality, level of duplications, GC-content, k-mer content and adapter contamination. Duplicate ChIP-seq reads or reads with a quality <30 were subsequently removed using Samtools with the “fixmate -m” and “markdup -r” tools.
BrU-seq reads were pre-filtered by alignment to the human ribosomal repeating subunit (GenBank U13369.1) and human mitochondrial genome (chrM) from the hg38 reference genome using bowtie2 (Version 2.3.3.1). The remaining reads were mapped to the hg38 reference genome using STAR (Version 2.7.0f).
Quality of obtained results were further validated by performing replicate experiments and experiments with different UV-C doses, and by testing the reversal of phenotypes over time.

Software

FastQC (Version 0.11.2), BWA (Version 0.7.16a), IGV (Version 2.4.3), MACS2 (Version 2.1), Samtools (Version 1.6), HOMER (Version 4.8.2), R (Version 3.5.3) and Rstudio (Version 1.1.423).
